# Genome-wide association study in Turkish and Iranian populations identify rare familial Mediterranean fever gene (MEFV) polymorphisms associated with ankylosing spondylitis

**DOI:** 10.1371/journal.pgen.1008038

**Published:** 2019-04-04

**Authors:** Zhixiu Li, Servet Akar, Handan Yarkan, Sau Kuen Lee, Pınar Çetin, Gerçek Can, Gökce Kenar, Fernur Çapa, Omer Nuri Pamuk, Yavuz Pehlivan, Katie Cremin, Erika De Guzman, Jessica Harris, Lawrie Wheeler, Ahmadreza Jamshidi, Mahdi Vojdanian, Elham Farhadi, Nooshin Ahmadzadeh, Zeynep Yüce, Ediz Dalkılıç, Dilek Solmaz, Berrin Akın, Salim Dönmez, İsmail Sarı, Paul J. Leo, Tony J. Kenna, Fatos Önen, Mahdi Mahmoudi, Matthew A. Brown, Nurullah Akkoc

**Affiliations:** 1 Translational Genomics Group, Institute of Health and Biomedical Innovation, Queensland University of Technology at Translational Research Institute, Princess Alexandra Hospital, Brisbane, Australia; 2 Department of Internal Medicine, Division of Rheumatology, Izmir Katip Çelebi University School of Medicine, İzmir, Turkey; 3 Department of Internal Medicine, Division of Rheumatology, Dokuz Eylul University, Faculty of Medicine, İzmir, Turkey; 4 Department of Molecular Medicine, Dokuz Eylul University Health Sciences Institute, İzmir, Turkey; 5 Department of Internal Medicine, Division of Rheumatology, Trakya University Medical Faculty, Edirne, Turkey; 6 Department of Rheumatology, School of Medicine, Uludağ University, Bursa, Turkey; 7 The University of Queensland Diamantina Institute, Translational Research Institute, Brisbane, Australia; 8 Rheumatology Research Center, Tehran University of Medical Sciences, Tehran, Iran; 9 Department of Internal Medicine, Division of Rheumatology, Celal Bayar University, Manisa, Turkey; The Walter and Eliza Hall Institute, AUSTRALIA

## Abstract

Ankylosing spondylitis (AS) is a highly heritable immune-mediated arthritis common in Turkish and Iranian populations. Familial Mediterranean Fever (FMF) is an autosomal recessive autoinflammatory disease most common in people of Mediterranean origin. *MEFV*, an FMF-associated gene, is also a candidate gene for AS. We aimed to identify AS susceptibility loci and also examine the association between *MEFV* and AS in Turkish and Iranian cohorts. We performed genome-wide association studies in 1001 Turkish AS patients and 1011 Turkish controls, and 479 Iranian AS patients and 830 Iranian controls. Serum IL-1β, IL-17 and IL-23 cytokine levels were quantified in Turkish samples. An association of major effect was observed with a novel rare coding variant in *MEFV* in the Turkish cohort (rs61752717, M694V, OR = 5.3, *P =* 7.63×10^−12^), Iranian cohort (OR = 2.9, *P =* 0.042), and combined dataset (OR = 5.1, *P =* 1.65×10^−13^). 99.6% of Turkish AS cases, and 96% of those carrying *MEFV* rs61752717 variants, did not have FMF. In Turkish subjects, the association of rs61752717 was particularly strong in *HLA-B27*-negative cases (OR = 7.8, *P* = 8.93×10^−15^), but also positive in *HLA-B27*-positive cases (OR = 4.3, *P* = 7.69×10^−8^). Serum IL-1β, IL-17 and IL-23 levels were higher in AS cases than controls. Among AS cases, serum IL-1β and IL-23 levels were increased in *MEFV* 694V carriers compared with non-carriers. Our data suggest that FMF and AS have overlapping aetiopathogenic mechanisms. Functionally important *MEFV* mutations, such as M694V, lead to dysregulated inflammasome function and excessive IL-1β function. As IL-1 inhibition is effective in FMF, AS cases carrying FMF-associated *MEFV* variants may benefit from such therapy.

## Author summary

Ankylosing spondylitis (AS) is a highly heritable immune-mediated arthritis. To identify new genetic associations with AS, we performed genome-wide association studies in Turkish and Iranian AS patients and controls. We identified a novel rare coding *MEFV* variant associated with AS. Rare polymorphisms of *MEFV*, which encodes the protein pyrin, are known to cause Familial Mediterranean Fever (FMF), a monogenic, autosomal recessive, autoinflammatory disease which can be complicated by arthritis. 99.6% of Turkish AS cases, and 96% of those carrying the *MEFV* variant, did not have FMF, and the association with AS remains excluding cases with FMF. In Turkish subjects, the *MEFV* variant association was particularly strong in *HLA-B27*-negative cases, but also positive in *HLA-B27*-positive cases. This represents the first rare variant association with AS, and has the highest odds ratio for AS of any non-MHC reported hitherto, indicating a major effect on disease pathogenesis. We assessed serum cytokine levels in the cohort, and found that IL-1β, IL-17 and IL-23 levels were higher in AS cases. Furthermore, among AS cases, IL-1β and IL-23 levels were increased in *MEFV* variant carriers compared with non-carriers. This study has therapeutic implications; as IL-1 inhibition is effective in FMF, AS cases carrying FMF-associated *MEFV* variants may benefit from such therapy.

## Introduction

Ankylosing spondylitis (AS) is a common form of arthritis affecting primarily the spine and pelvic sacroiliac joints. Twin studies indicate that the disease is highly heritable, with over 90% of the risk of developing the condition being genetically determined [1, 2]. To date 114 loci have been identified as being associated with the disease, contributing roughly 30% of the overall risk [3]. The most strongly associated variants at these loci are all common (minor allele frequency (MAF)>5%) or low-frequency (MAF 1–5%) variants, and no rare variant (MAF<1%) has yet been demonstrated to be AS-associated at genome-wide significance (*P*<5×10^−8^).

AS is found in most ethnic groups with the notable exceptions of Africans and Australian Aboriginals, in whom the prevalence of *HLA-B27* is very low, or as yet unidentified environmental factors protect against disease development. No GWAS has been performed to date in AS cases of Turkish or Iranian descent. These groups are of particular interest because of evidence that patients with the monogenic autoinflammatory disease, Familial Mediterranean Fever (FMF), have an increased prevalence of sacroiliitis [4–9], and that FMF-unaffected first-degree relatives of FMF patients have an increased frequency of AS [5], suggesting an aetiopathogenic link with AS. There have also been four candidate gene case-control association studies of *MEFV* variants in AS, three of which have demonstrated nominal (10^−5^<*P*<0.05) association of the main FMF-associated *MEFV*-variant (rs61752717, M694V) with AS [10–13], with one marginally negative study (*P =* 0.065) [14]. All of the participants in these studies were from Turkey. However, the sample sizes of these studies were relatively small (number of patients ranging from 62 to 193) and, therefore, lacked power to achieve definitive significance.

In order to identify new genetic variants associated with AS, and to investigate the potential association of *MEFV* variants in AS, we performed a genome-wide association study in case-control cohorts from Turkey and Iran.

## Results

### AS-Associated loci in Turkish and Iranian GWAS cohorts

As expected, SNPs in the major histocompatibility complex on chromosome 6p21, and imputed *HLA*-alleles, were strongly associated with AS in both the Turkish and Iranian cohorts (Table 1). In the Turkish cohort the strongest SNP associations were with genotyped SNP rs17192932 (OR = 22.84, 95% CI = 17.55–29.72, *P =* 5.64×10^−120^), and in the Iranian cohort with rs117486637 (OR = 31.08, 95% CI = 22.27–43.38, *P =* 8.62×10^−91^). The strength of association of *HLA-B27* with AS was similar in both Turkish and Iranian cohorts, but the proportion of *HLA-B27* carriers was lower than in most studies of European descent AS cases (71% and 74%, respectively, compared with 80–95% in most European descent cohorts), consistent with previous reports. Stepwise conditional analyses confirmed risk associations with *HLA-B40* subtypes in both cohorts, with HLA-B*5101 in the Turkish cohort, and with HLA-B*1503 in the Iranian cohort. HLA-B*5101 also achieved nominal significance in the Iranian cohort (OR = 1.51, 95%CI = 1.055–2.15, *P* = 0.024).

**Table 1:** Stepwise conditional logistic regression analysis of HLA-B allelic associations with ankylosing spondylitis in the Turkish and Iranian cohorts.

Turkish Cohort					
Round	HLA-B allele	OR (95% CI)	Stepwise conditional *P*-value	Initial *P*-value	Allele frequency (case/control)
1	*2705	34.31 (25.33–46.48)	1.31×10^−88^	1.31×10^−88^	0.311/0.026
2	*2702	44.93 (19.27–104.7)	1.23×10^−18^	4.48×10^−11^	0.049/9.003
3	*2704	15.94 (3.461–73.37)	0.00038	0.041	0.005/0.001
4	*5101	1.61 (1.241–2.089)	0.00034	0.15	0.1/0.117
5	*4006	3.06 (1.419–6.613)	0.0044	0.75	0.009/0.008
Iranian Cohort					
Round	HLA-B allele	OR (95% CI)	Stepwise conditional *P*-value	Initial *P*-value	
1	*2705	57.54 (38.17–86.74)	9.65×10^−72^	9.65×10^−72^	0.345/0.017
2	*2707	15.84 (4.97–50.53)	3.05×10^−6^	0.0059	0.013/0.003
3	*2704	21.3 (4.37–103.8)	0.00015	0.021	0.008/0.001
4	*4002	8.03 (2.14–30.06)	0.0020	0.036	0.008/0.001
5	*1503	8.22 (2.17–31.09)	0.0019	0.23	0.006/0.003
6	*2702	27.28 (3.02–246.5)	0.0032	0.077	0.005/0.0007

CI, confidence interval; OR, odds ratio.

Association at genome-wide significance (*P*<5×10^−8^) was observed with two non-MHC loci in the Turkish cohort (Table 2), including the known AS-associated locus *USP8*, and the *MEFV* locus, and with one known AS-associated locus in the Iranian cohort (*ERAP1*; Table 3).

**Table 2:** Genome-wide significant and suggestive non-MHC loci for ankylosing spondylitis in the Turkish cohort.

Chr	Pos^a^	SNP	*P*-value	OR (95%CI)	Nearby genes	Risk /Prot	MAF Case /Con
4	11,504,491	rs10000518	6.05×10^−6^	0.48(0.35–0.66)	*HS3ST1*	G/A	0.03/0.07
4	23,346,184	rs34939008	7.20×10^−6^	0.74(0.65–0.84)	*GBA3*	G/A	0.39/0.46
7	38,706,817	rs10280089	1.08×10^−6^	1.44(1.24–1.67)	*FAM183B*	A/C	0.32/0.24
8	2,139,471	rs4876208	5.56×10^−6^	1.37(1.20–1.57)	*MYOM2*	C/G	0.39/0.32
8	24,116,722	rs1013210	7.88×10^−8^	0.65(0.56–0.76)	*ADAM28*	G/A	0.20/0.28
*15*	*50*,*785*,*016*	*rs148783236*^b^	*1*.*01×10*^*−11*^	*0*.*52(0*.*43–0*.*63)*	*USP8*	*A/G*	*0*.*12/0*.*20*
15	70,338,485	rs11639037	9.52×10^−6^	1.40(1.21–1.62)	*TLE3*	C/T	0.31/0.24
15	101,767,822	rs8036083	7.24×10^−6^	1.35(1.19–1.55)	*CHSY1*	G/T	0.47/0.40
*16*	*3*,*293*,*407*	*rs61752717*	*7*.*63×10*^*−12*^	*5*.*34(3*.*31–8*.*62)*	*MEFV*	*C/T*	*0*.*06/0*.*01*
*16*	*3*,*293*,*407*^c^	*rs61752717*	*1*.*75×10*^*−11*^	*5*.*26 (3*.*24–8*.*54)*	*MEFV*	*C/T*	*0*.*058/0*.*01*
18	14,723,700	chr18:14723700:D	8.78×10^−6^	0.74(0.65–0.84)	*ANKRD30B*	Y/Z	0.36/0.43
21	46,272,105	rs235316	7.85×10^−6^	1.44(1.23–1.69)	*PTTG1IP*	T/A	0.25/0.19

Genome-wide significant loci details appear in italics. All others had suggestive significance.

Chr, chromosome; CI, confidence interval; Con, control; MAF, minor allele frequency; OR, odds ratio; Pos, position; Prot, protective.

^a^Human genome build hg19

^b^rs148783236 is exm1161045 in the raw data

^c^excluded four FMF cases

**Table 3:** Genome-wide significant and suggestive non-MHC loci for ankylosing spondylitis in the Iranian cohort.

Chr	Pos^a^	SNP	*P*-value	OR (95% CI)	Nearby genes	Risk /Prot	MAF Case/Con
2	62568221	rs13001372	5.33×10^−7^	1.56 (1.31–1.85)	*2p15*	A/G	0.49/0.40
*5*	*96126308*	*rs27529*	*1*.*12×10*^*−9*^	*1*.*74 (1*.*46–2*.*08)*	*ERAP1*	*A/G*	*0*.*48/0*.*39*
9	78503158	rs7874251	6.35×10^−6^	0.66 (0.55–0.79)	*PCSK5*	A/G	0.33/0.42
10	90754487	rs2031610	4.67×10^−6^	1.49 (1.26–1.77)	*FAS*	C/T	0.50/0.40
12	26043525	rs7298011	4.11×10^−6^	1.62 (1.32–1.99)	*RASSF8*	A/G	0.25/0.17
19	17451098	rs4808624	9.99×10^−6^	1.48 (1.24–1.75)	*DDA1*	G/C	0.47/0.44

Genome-wide significant loci details appear in italics. All others had suggestive significance.

Chr, chromosome; CI, confidence interval; Con, control; MAF, minor allele frequency; OR, odds ratio; Pos, position; Prot, protective.

^a^Human genome build hg19

No additional loci were identified at genome-wide significance in the meta-analysis combining both cohorts. In the meta-analysis the strongest non-MHC association was with SNPs in the *USP8* locus, which has a strong protective effect in AS (exm1161045/rs148783236, OR = 0.58, *P* = 1.43×10^−13^). The novel *MEFV* variant, rs61752717, is also identified at genome-wide significance in the meta-analysis. Genome-wide significant association was also replicated with SNPs in chromosome 2p15 (2p15) and *ERAP1* (Table 4). In each of these cases, the most strongly associated SNP is in strong linkage disequilibrium (*r*^2^>0.8) with the previously reported AS-associated variant in European descent populations. Only nominal significance was seen with SNPs at *IL23R*, a known strongly AS-associated locus, in either dataset or in the meta-analysis analysis. There were 105 out of 113 non-MHC tag SNPs for known AS-associated loci in the combined cohort, while eight of them were missing in the dataset. Two of them (*2p15* and *ERAP1*) were GWS in the combined cohort. One was at suggestive significance (*NOS2*). Twenty-one of them were at nominal significance. All these 24 tag SNPs were in the same direction of effect as they were in the cross-disease study.

**Table 4:** Genome-wide significant and suggestive non-MHC loci for AS in the meta-analysis of both Iranian and Turkish cohorts. The loci in italics were in the known 113 loci.

Chr	Pos^a^	SNP	*P*-value	OR (95%CI)	Nearby genes	Risk /Prot
*2*	*62*,*568*,*221*	*rs13001372*	*7*.*23×10*^*−9*^	*1*.*36 (1*.*19–1*.*56)*	*2p15*	*A/G*
4	11,504,491	rs10000518	6.05×10^−6^	0.48 (0.35–0.66)	*HS3ST1*	G/A
4	23,346,184	rs34939008	7.19×10^−6^	0.74 (0.65–0.84)	*GBA3*	G/A
*5*	*96*,*126*,*308*	*rs27529*	*1*.*36×10*^*−9*^	*1*.*39 (1*.*22–1*.*59)*	*ERAP1*	*A/G*
7	38,706,817	rs10280089	1.1×10^−6^	1.44 (1.24–1.67)	*AMPH*	A/C
8	2,139,471	rs4876208	5.62×10^−6^	1.37 (1.20–1.57)	*MYOM2*	C/G
8	24,116,722	rs1013210	7.85×10^−8^	0.65 (0.56–0.76)	*ADAM28*	G/A
9	78,503158	rs7874251	6.34×10^−6^	0.66 (0.55–0.79)	*PCSK5*	G/A
10	90,754,487	rs2031610	4.6×10^−6^	1.49 (1.26–1.77)	*FAS*	C/T
12	26,043,525	rs7298011	4.07×10^−6^	1.62 (1.32–1.99)	*RASSF8*	A/G
*15*	*50*,*785*,*016*	*rs148783236*	*1*.*44×10*^*−13*^	*0*.*58 (0*.*48–0*.*69)*	*USP8*	*A/G*
15	70,338,485	rs11639037	9.71×10^−6^	1.40 (1.20–1.62)	*TLE3*	C/T
15	101,767,822	rs8036083	7.37×10^−6^	1.35 (1.19–1.55)	*CHSY1*	G/T
*16*	*3*,*293*,*407*	*rs61752717*	*1*.*72×10*^*−12*^	*4*.*76 (2*.*94–7*.*68)*	*MEFV*	*C/T*
17	147,35,492	rs6502398	5.18×10^−7^	0.76 (0.67–0.87)	*17p12*	C/A
17	260,98,781	rs7217335	1.34×10^−6^	1.34 (1.15–1.56)	*NOS2*	A/G
18	1,4723,700	chr18:14723700:D	8.79×10^−6^	0.74 (0.65–0.84)	*ANKRD30B*	Y/Z
19	17451098	rs4808624	9.99×10^−6^	1.48 (1.24–1.75)	*GTPBP3*	G/C
21	46,272,105	rs235316	7.87×10^−6^	1.44 (1.23–1.69)	*PTTG1IP*	T/A

Genome-wide significant loci details appear in italics. All others had suggestive significance.

Chr, chromosome; CI, confidence interval; Con, control; MAF, minor allele frequency; OR, odds ratio; Pos, position; Prot, protective.

^a^Human genome build hg19

Suggestive associations (5×10^−8^ <*P*<1×10^−5^) were observed at nine novel loci in the Turkish cohort, and three novel and two known AS-associated loci (2p15 and *FAS*) in the Iranian cohort (Tables 2 and 3, S1–S20 Figs). In the Turkish cohort, near genome-wide significant association was seen with SNPs in *ADAM28* (encoding ADAM metallopeptidase domain 28; rs1013210, OR = 0.65, 95% CI = 0.56–0.76, *P =* 7.88×10^−8^). No association was seen at this locus in the Iranian cohort despite the MAF being similar in Turkish and Iranian controls (MAF = 0.28 and 0.25, respectively).

In the Turkish cohort, the strongest *MEFV* association observed was with the non-synonymous SNP rs61752717 (OR = 5.34, 95% CI = 3.31–8.62, *P* = 7.63×10^−12^), which encodes the M694V protein variant which is the most strongly associated FMF allele. Excluding four cases who have co-existent AS and FMF, this association remains genome-wide significant (OR = 5.26, 95% CI = 3.24–8.54, *P* = 1.75×10^−11^). This polymorphism was also nominally associated with AS in the Iranian cohort (OR = 2.85, 95% CI = 1.039–7.83, *P* = 0.042), its MAF being 3.3-times lower than in the Turkish controls (MAF = 0.0033 in Iranian controls, 0.011 in Turkish controls; Table 5). In the meta-analysis, association was seen with OR = 4.76, 95% CI = 2.94–7.68, *P* = 1.72×10^−12^. Nominal association was seen with multiple other *MEFV* SNPs in both the Turkish and Iranian cohorts, but none of these are considered FMF-associated, and none were shared between the two cohorts (S1 Table).

**Table 5:** Comparison of *MEFV* M694V in Turkish, Iranian and the meta-analysis of both cohorts.

rs61752717	Case maf	Control maf	#cases	#controls	OR	*P*-value
Turkish	0.061	0.011	921	907	5.34	7.63×10^−12^
Iranian	0.012	0.0033	422	754	2.85	0.042
meta-analysis	0.047	0.0078	1344	1369	4.76	1.72×10^−12^

OR, odds ratio. maf, minor allele frequency.

### Interaction analysis between *MEFV* variants and HLA-B27

Previous studies have suggested interaction between *HLA-B27* and *MEFV* in AS (OR = 8.69 in *HLA-B27*-positive, and 21.8 in *HLA-B27*-negative cases) [11]. In the current study, rs61752717 shows stronger association in *HLA-B27*-negative patients (*P* = 8.93×10^−15^) than in *HLA-B27*-positive patients against all controls (*P* = 7.69×10^−8^, Table 6). In the Turkish cohort, in comparison of cases alone, rs61752717 risk allele carriage was higher in *HLA-B27*-negative than positive cases (OR = 2.67, 95% CI = 1.78–4.01, *P =* 1.28×10^−6^ in whole cases cohort, OR = 2.39, CI = 1.58–3.64, *P =* 9.54×10^−6^ excluding four cases with co-existent FMF and AS, Fisher Exact Test, Table 6), whereas no difference was observed in controls (*P* = 0.25). Compared with all-Turkish controls, the association of rs61752717 was much stronger in *HLA-B27*-negative cases (OR = 7.8, *P* = 8.93×10^−15^ in whole cohort, OR = 7.6, *P* = 3.92×10^−14^ excluding four cases with co-existent FMF and AS) than in *HLA-B27*-positive cases (OR = 4.3, *P* = 7.69×10^−8^), although no interaction observed in a specific logistic regression analysis (*P =* 0.97). Similarly, rs61752717 risk allele carriage was higher in *HLA-B27*-negative than positive cases in the Turkish-Iranian combined cohort (OR = 2.74, 95% CI = 1.86–4.03, *P* = 1.29×10^−7^ in entire cohort; OR = 2.47, 95% CI = 1.66–3.67, *P* = 5.78×10^−6^ excluding four cases with co-existent FMF and AS; Table 7). However, no significant difference was observed in rs61752717 risk allele carriage between *HLA-B27* positive and *HLA-B27*-negative patients (OR = 1.44, 95% CI = 0.23–6.40, *P* = 0.70; Table 8) in the Iranian cohort, noting the extremely low power of this analysis given the rarity of rs61752717 in this cohort.

**Table 6:** Genotype counts and (%) of rs61752717 in *HLA-B27*-positive and *HLA-B27*-negative Turkish cohorts (including four HLA-B27-negative subjects with co-existent AS and FMF, three homozygous for rs61752717 ‘C’ allele, one homozygote for rs61752717 ‘T’ allele).

rs61752717	Case		Control		Total
	B27+	B27−	B27+	B27−	
CC (+/+)	1 (0.2)	9 (3.4)	0 (0)	0 (0)	10
CT (+/−)	53 (8.2)	38 (14.3)	0 (0)	19 (2.3)	110
TT (−/−)	596 (91.7)	218 (82.3)	57 (100)	824 (97.7)	1695
CC or CT (+/+ or +/−)	54 (8.3)	47 (17.7)	0 (0)	19 (2.3)	120
Sum	650	265	57	843	1815

rs61752717 genotype counts differ between HLA-B27-positive and–negative cases (*P =* 1.22×10^−6^). Comparing allele frequencies between HLA-B27-positive and–negative controls, no difference was observed (CC genotype frequency comparison not possible due to zero counts of CC genotypes; *P =* 0.25). Risk allele carriage in *HLA-B27*-negative cases was higher than in positive cases (OR = 2.67, 95% CI = 1.78–4.01, *P =* 1.28×10^−6^ in whole cases cohort, OR = 2.39, 95% CI = 1.58–3.64, *P =* 9.54×10^−6^ excluding four cases with co-existent FMF and AS).

**Table 7:** Genotype counts and (%) of rs61752717 in *HLA-B27*-positive and *HLA-B27*-negative combined cohorts (including four HLA-B27-negative subjects with co-existent AS and FMF, three homozygous for rs61752717 ‘C’ allele, one homozygote for rs61752717 ‘T’ allele).

rs61752717	Case		Control		Total
	B27+	B27−	B27+	B27−	
CC (+/+)	1 (0.1)	11 (3.0)	0 (0)	0 (0)	12
CT (+/−)	57 (6.1)	39 (10.6)	0 (0)	24 (1.6)	120
TT (−/−)	869 (97.3)	319 (86.4)	90 (100)	1479 (98.4)	2757
CC or CT (+/+ or +/−)	58 (6.3)	50 (13.6)	0 (0)	24 (1.6)	132
Sum	927	369	90	1503	2889

Risk allele carriage in *HLA-B27*-negative cases was higher than positive cases (OR = 2.74, 95% CI = 1.86–4.03, *P =* 1.29×10^−7^ in whole cases cohort, OR = 2.47, 95% CI = 1.66–3.67 *P =* 5.78×10^−6^ excluding four cases with co-existent FMF and AS).

**Table 8:** Genotype counts and (%) of rs61752717 in *HLA-B27*-positive and *HLA-B27*-negative Iranian cohorts.

rs61752717	Case		Control		Total
	B27+	B27−	B27+	B27−	
CC (+/+)	0 (0)	2 (1.8)	0 (0)	0 (0)	2
CT (+/−)	5 (1.5)	1 (0.9)	0 (0)	5 (0.7)	11
TT (−/−)	299 (98.4)	111 (97.4)	35 (100)	710 (99.3)	1155
CC or CT (+/+ or +/−)	5 (1.5)	3 (2.6)	0 (0)	5 (0.7)	13
Sum	304	114	35	715	1168

No significant difference was observed in risk allele carriage between *HLA-B27*-negative cases and positive cases (OR = 0.37, 95% CI = 0.084–1.623, *P* = 0.14)

### Interaction analysis between *MEFV* variants and HLA-B51

The *MEFV* nsSNP rs61752717 is also associated with Behçet’s Disease (BD) [15–18]. Joint involvement is common in BD, and about 5% of the BD patients developed sacroiliitis [19]. The major genetic association with BD is with *HLA-B51* [20], which is also AS-associated [21]. rs61752717 was also more strongly associated with AS in *HLA-B51*-negative than in *HLA-B51*-positive restricted analyses (OR = 5.88, 95% CI = 3.31–10.42, *P* = 1.36×10^−9^ vs OR = 4.82, 95% CI = 1.72–11.66, *P* = 2.11×10^−3^, or OR = 5.88, 95% CI 3.32–10.43, *P* = 1.33×10^−9^ vs OR = 4.24, 95% CI 1.58–11.42, *P* = 4.22×10^−3^ if excluding four cases with co-existent FMF and AS; S2 Table) but the difference was not statistically significant, nor was interaction observed in a specific logistic regression analysis (*P =* 0.69). No significant difference was observed in rs61752717 risk allele carriage between *HLA-B51-*positive and *HLA-B51*-negative patients (OR = 1.44, 95% CI = 0.23–6.40, *P* = 0.70) in the Iranian cohort. Similarly, there was no significant interaction identified in the Iranian cohort (*P* = 0.975; S3 Table) nor in the combined cohort (*P* = 0.994; S4 Table).

#### Serum IL-1β and IL-23 levels were significantly higher in *MEFV* 694V variant carriers

*MEFV* encodes the protein pyrin which functions to control caspase-1-induced IL-1β production. FMF-associated *MEFV* variants are known to lead to excess IL-1β production, and IL-1 inhibition is effective in treatment of FMF and in a subset of AS patients [22]. Previous studies have also suggested the involvement of Th17 lymphocytes in FMF [23, 24]. We therefore compared serum IL-1β, IL-17 and IL-23 cytokine levels in *MEFV* risk variant carriers in Turkish AS cases and healthy controls. Levels of all three cytokines were significantly elevated in cases compared with controls (Table 9). Among AS cases, serum IL-1β and IL-23 levels were significantly increased in *MEFV* 694V variant carriers compared with non-carriers (*P* = 0.0017 and 0.0068, respectively). The result is similar after removing the subjects with co-existent AS and FMF.

**Table 9:** Serum cytokine levels by case or control status and *MEFV* M694V carrier status.

Cytokine	Control (mean (SE))	Case (mean (SE))	Case-control *P*-value	*MEFV* 694M Carrier	*MEFV* 694V Carrier	Genotype *P*-value
IL-1	0.19 (0.042)	1.98 (0.39)	0.00030	0.95 (0.21)	3.39 (0.83)	0.0017
IL-1*		2.16 (0.70)	0.00041	0.93 (0.21)	3.43 (0.86)	0.0093
IL-17	0.41 (0.086)	2.21 (0.68)	0.033	1.96 (0.90)	2.56 (1.06)	0.89
IL-17*		1.95 (0.41)	0.016	1.99 (0.92)	2.39 (1.11)	0.78
IL-23	8.20 (3.85)	26.90 (4.47)	0.0036	16.72 (3.86)	40.81 (8.63)	0.0068
IL-23*		28.09 (4.61)	0.0023	17.14 (3.93)	43.72 (9.00)	0.0038

Cytokine levels are expressed as pg/mL.

*excluding the subjects with co-existent AS and FMF.

Note that the carrier *MEFV* M694V status is for cases only.

## Discussion

This study confirms that *MEFV* is associated with both *HLA-B27*-positive and–negative AS, even in the absence of clinical evidence of FMF. The association represents the first rare variant association with AS, and has the highest odds ratio for disease of any non-MHC reported locus to date, indicating a major effect on disease pathogenesis. The strength of association observed in the Turkish and Iranian populations was, as expected, closely related to the MAF of the polymorphisms involved, and therefore the absence of association of *MEFV* with AS in previous studies of other populations may simply due to ethnic differences in gene frequencies rather than differences in the underlying mechanisms driving disease. Further studies examining, for example, gene-expression in patients and healthy controls and in relation to disease activity, will be required to determine whether MEFV-driven autoinflammatory processes are a factor in AS in patients in populations where MEFV functional variants are much rarer.

The strength of the association of the M694V MEFV polymorphism with AS is much stronger in *HLA-B27*-negative than–positive AS. Whilst a formal test of interaction between *HLA-B27* and rs61752717 was negative, the stronger association in *HLA-B27*-negative cases is consistent with previous studies [11], and suggests that this is the third example of epistasis found in AS genetics, following the previously demonstrated examples of *ERAP1* variants and *HLA-B27* and *HLA-B40*. The strength of association in *HLA-B27*-negative subjects is considerable and is to our knowledge the strongest effect in terms of odds ratio of any non-MHC variant in a common immune-mediated disease. In the absence of a definite explanation as to how HLA-B27 induces AS, it is not possible to provide a functional explanation for to this finding. However, one possible hypothesis is that HLA-B27-positive disease is primarily driven by different immunological pathways than HLA-B27-negative MEFV-positive disease.

The replication of the association of *HLA-B51* with AS, previously observed in western European-descent AS [21], in the Turkish and Iranian cohorts in the current study also provides further support that AS and Behcet’s disease have overlapping aetiopathogenesis. Behçet’s disease is also associated with *MEFV* polymorphisms, and Behçet’s disease cases carrying *MEFV* variants have more severe disease [15–17], suggesting that autoinflammation may also contribute to its development. This study also provides further confirmation of the association of *HLA-B40* alleles with AS [25, 26]. The study sample size was too small to test whether gene-gene interaction with *ERAP1* variants was observed, as has previously been demonstrated in AS cases and controls of western European-descent with both *HLA-B27* and *HLA-B40* [21].

We also demonstrated that serum IL-1β levels are higher in Turkish AS cases than healthy controls, and are higher in carriers of the *MEFV* M694V polymorphism. Pyrin, encoded by *MEFV*, has been shown to negatively or positively regulate caspase-1 and IL-1β activation, depending on the experimental system used [27–29]. In a loss of function model, *MEFV* variants operate by attenuating the apoptosis-associated speck-like protein containing a caspase recruitment domain (ASC, encoded by *Pycard)* -dependent and ASC independent inhibitory effects of pyrin on caspase activation and subsequent IL-β production [27, 28], whereas in a gain-of-function model they lead to caspase-1 activation through an ASC-dependent, NLRP3-independent, pyrin inflammasome [29]. In either model *MEFV* variants lead to excessive IL-1β production.

Pyrin is also a member of the tripartite motif (TRIM) family proteins, which are critically involved in regulation of autophagy and innate immunity. Pyrin/TRIM20, not only acts as a specific receptor for NLRP1, NLRP3 and pro-caspase 1, but also serves as a platform for assembly of key regulators (ULK1, Beclin1, and ATG16L1) and effector factors (mAtg8s) of autophagy initiation machinery [30]. Through these abilities, pyrin leads to degradation of key inflammasome targets in a highly selective manner, resulting in suppression of caspase-1 activation and IL-1β production. Pyrin variants harboring FMF-associated B30.2 mutations, including the M694V variant, have been shown to be associated with a deficiency in the autophagic degradation of NLRP3 [30]. This finding adds more to the complexity and multiplicity of possible mechanisms underlying the excessive IL-1β production seen in patients with FMF and many other autoinflammatory diseases. Pyrin/TRIM20, whose expression is significantly up-regulated by IFN-gamma [29], is one of the required mediators of IFN-gamma-induced autophagy [30], providing further support for pyrin’s regulating role in restraining excessive inflammation induced by innate immunity.

In the light of above-mentioned findings, increased M694V prevalence in AS patients observed in our study adds to the mounting evidence that AS is an autoinflammatory disease [31], and also provides support to the recent hypothesis that autophagy may be involved in AS pathogenesis [32]. In the latter context, it is worth mentioning a recent study that showed decreased expressions of autophagy-related genes (*MAP1LC3A*, *BECN1*, and *ATG5*) in the peripheral blood mononuclear cells of AS patients as compared to controls [33]. Notably, even further decreased levels of expression of *MAP1LC3A* and *BECN1* were found in patients with more advanced spinal damage in the same study [33]. Further, there is substantial evidence of activation of the innate immune system in AS, with studies demonstrating that ILC3, MAIT and γδ cells are major sources the pro-inflammatory cytokines IL-17 and IL-22 in the disease [34–36], consistent with a role for autoinflammation in AS pathogenesis.

Previous AS genetic studies have demonstrated genome-wide significant associations of the IL-1 receptor genes, *IL1R2* and *IL1R1* [37], further supporting involvement of this cytokine pathway in the disease. Serum IL-1β levels have been shown previously not to be elevated in AS patients of western European descent [38, 39]. Similarly, no difference in serum IL-1β levels were seen in a previous study of Turkish AS cases and controls [40], although in this study no information is reported on the age and gender matching of the cases and controls. Put together, this evidence suggests that IL-1 related processes are important in AS in different ethnic populations, rather than being restricted to *MEFV* M694V carriers, but that in this group IL-1 plays a major immunopathogenic role. This may explain why minimal association was seen with *IL23R* variants in this population, perhaps suggesting that the IL-23 cytokine pathway is less important in this group of patients, where IL-1 effects may predominate.

The low efficacy of IL-1 blockade with the IL-1 receptor antagonist anakinra in AS suggests that the disease, at least in the western European population studies, is driven by factors largely independent of IL-1 [22, 41]. In contrast, IL-1 blockade is highly effective for FMF caused by the same *MEFV* polymorphism we demonstrate here to be associated with AS. Our genetic and cytokine level findings support the hypothesis that, at least in patients with the M694V *MEFV* variant, IL-1 blockade is a potential worthwhile therapeutic option. Anakinra has been shown to be effective in relieving signs and symptoms of spondyloarthritis associated with FMF [42–44]. Of particular interest, one of these cases had severe axial pain and elevated acute phase markers persisting despite treatment courses with adalimumab and etanercept, but both the clinical symptoms and acute phase reactant levels improved dramatically with IL-1 blocking therapy [44].

Bacterial pathogens are thought to also be drivers of inflammation in AS, as suggested by microbiome studies demonstrating differences in the gut microbiome in AS cases and controls [45], and the association of the bacterial lipopolysaccharide innate immune receptor *TLR4* variants with AS [3]. *MEFV* variants, and particularly the M694V polymorphism, are also associated with both ulcerative colitis and Crohn’s disease in Turkish patients, suggesting a particular role for MEFV in gut mucosal inflammation [46, 47]. FMF patients have been shown to have intestinal microbiome dysbiosis, both whilst in remission and during attacks, consistent with models of FMF whereby gut bacterial interactions with a genetically primed host immune system drive disease. At genus level resolution, similarities between what was seen in FMF patients and AS cases are striking, with increases in carriage of *Ruminococcus*, *Porphyromonadaceae*, and reductions in *Prevotella* seen in both diseases [45, 48]. Further studies in AS and FMF cases matched for ethnicity and other relevant covariates would appear indicated to determine if specific bacterial species or components are common drivers or have protective effects in these two diseases.

In conclusion, this study demonstrates that the *MEFV* M694V (rs61752717) SNP markedly increases the risk of AS even in patients not suffering from FMF, and is associated with increased serum IL-1β levels in those patients, suggesting that IL-1 inhibition may be effective in that subset of AS patients, and that MEFV-driven autoinflammation is a factor in the aetiopathogenesis of AS.

## Materials and methods

### Ethics statement

All case and control participants gave written, informed consent. The study was approved by the relevant research ethics authorities at each participating centre, and overall approval was given by Queensland University of Technology Health Research Ethics Committee (approval number HREC/05/QPAH/221).

### Study participants

AS was defined according to the modified New York criteria. In total, the study involved 1001 Turkish AS patients, 1011 Turkish controls, 479 Iranian AS patients and 830 Iranian controls. All cases were specifically screened by recruiting clinicians for a history of FMF.

### Genotyping

DNA from all subjects were genotyped using the Illumina CoreExome chip following standard protocols at the Australian Translational Genomics Centre, Princess Alexandra Hospital, Brisbane. All Turkish samples were genotyped by CoreExome chips v1.0. 479 Iranian AS cases and 197 Iranian controls were genotyped by CoreExome chips v1.0, while the remaining 633 Iranian controls were genotyped by CoreExome chips v1.1. Bead intensity data was processed and normalized for each sample and genotypes called using the Illumina Genome Studio software.

A subset of 20 carriers of the *MEFV* M694V had the microarray genotypes tested by Sanger sequencing, with complete concordance of results.

### Data quality control

SNP genotypes from CoreExome v1.0 and v1.1 chips from the Iranian cohort were merged based on the manifest files and plink files. 349 SNPs were excluded due to uncertain strandedness or large difference (> 10%) in control allele frequencies between chip versions. We also removed the redundant SNPs with the same minor and major alleles in the same position but with different SNP IDs by keeping the one with lower sample missing rate. Quality control (QC) was completed separately on individual cohorts, and included assessment of missingness by individual (threshold <5%), missingness by genotype (threshold<5%), Hardy-Weinberg equilibrium in controls (*P* < 1×10^−6^), extreme heterozygosity (threshold > 3 standard deviations from mean) and identity by descent threshold at the inflation point of PI_HAT (0.2013 and 0.0981 for Turkish cohort and Iranian cohort, respectively). For each pair of related samples (PI_HAT > threshold), the sample with the higher missing rate was removed, and where the pair involved a case and a control with similar call rates (absolute difference in missing rate < 0.0005), cases were preferentially selected for inclusion.

Genotyped SNPs with MAF > 0.05 were then used to perform principal component analysis for ethnicity identification using SHELLFISH (http://www.stats.ox.ac.uk/~davison/software/shellfish/shellfish.php). Principal components analysis (PCA) was performed with 0–10 eigenvectors. Ethnic and ancestry outliers (more than 6 standard deviations from the mean on any principal component) were excluded. Ski-plots of λ and λ_1000_ for different numbers of eigenvectors as covariates for the Iranian, Turkish and combined cohorts are shown in S21 and S22 Figs, and plots of the principal components analysis (first vs second principal component) in S23–S25 Figs. For the Iranian cohort, adding any principal components increased the λ nor λ_1000_. The best λ was produced using no principal components (1.011, λ_1000_ = 1.022). In the Turkish cohort, use of the first five principal components produced λ at 1.022 (λ_1000_ = 1.024). Use of additional components did not reduce the λ nor λ_1000_ further. The quantile–quantile plots for the Iranian, Turkish and combined cohorts are shown in S26–S28 Figs, respectively.

After QC, there were 1,828 Turkish samples (921 cases and 907 controls) and 1,176 Iranian samples (422 cases and 754 controls). After SNP QC, there were total of 294,091 and 293,283 SNPs in the Turkish and Iranian cohorts, respectively.

### Imputation

Genotypes were imputed in candidate regions for both cohorts using the 1000 Genomes Project (Phase 1 integrated v3). Genotype data were phased with SHAPEIT [49], and genotypes were imputed with Impute2 [50]. SNPs with low imputation quality (*r*^2^ < 0.6) were excluded. HLA alleles and Amino Acid Polymorphisms in HLA region were imputed by SNP2HLA with T1DGC reference (collected by Type 1 Diabetes Genetics) [51].

### Association analysis

Logistic association analyses were performed using PLINK (v1.90b3.36, https://www.cog-genomics.org/plink2) [52] to perform with the first five principal components as covariates for population stratification control in the Turkish cohort. Covariates were not added in the Iranian cohort as there was little population stratification (with any covariates the λ increases). Genome-wide significance was accepted at *P*<5×10^−8^, and genome-wide suggestive at *P*<1×10^−5^. Association analysis in imputed genotypes was assessed with PLINK best guess genotypes.

Cluster plots for reported SNPs were checked manually in the cases and controls in the respective cohorts and with each different CoreExome chip version separately.

### Meta-analysis

The meta-analysis was performed by inverse-variance method implemented in the software package METAL [53].

### *HLA-B27*, *HLA-B51*, and rs61752717 interactions

Testing for interaction between *HLA-B51* and *HLA-B27* with the *MEFV* lead SNP rs61752717 in the corresponding cohort was performed by logistic regression fitting a dominant term for the respective HLA-B allele status (positive or negative) and an additive term for SNP rs61752717, including a multiplicative interaction term, and the N corresponding principal components for population stratification correction (N = 5, 0 and 4 for Turkish, Iranian and combined cohort, respectively):

$$Phenotype\;\sim \;rs61752717_{genotype}+HLA-Bstatus+HLA-Bstatus*rs61752717_{genotype}+PC1+\cdots +PCN$$

where *Phenotype* was coded as 2 (patient) and 1 (healthy control), *rs61752717 genotype was coded as 0 (genotype CC)*, *1 (genotype CT) and 2 (genotype TT) to* reflect additive effect, *HLA-B* allele status was coded as 0 or 1 to reflect dominant effect; *HLA* − Bstatus*rs61752717_*genotype*_ was the interaction term for *HLA-B* and rs61752717 genotype; PC*i* codes was the ith principal component from PCA from the corresponding cohort.

### Serum IL-1β, IL-17 and IL-23 cytokine levels

Concentrations of IL-1β and IL-17A in serum were measured using the Human IL-1β/IL-1F2 Quantikine HS ELISA kit (R&D Systems) and Human IL-17A High Sensitivity ELISA kit (Life Technologies) according to manufacturer’s instructions. Statistical significance of differences was evaluated using the Student’s *t*-test.

### Web resources

SHELLFISH; http://www.stats.ox.ac.uk/~davison/software/shellfish/shellfish.php

PLINK v1.90b3.36; https://www.cog-genomics.org/plink2

## Supporting information

S1 Table AS-associated variations in *MEFV* in Turkish and Iranian case-control cohorts separately. (DOCX)

S2 Table Genotype counts and (%) of rs61752717 in *HLA-B51*-positive and *HLA-B51*-negative cohorts in Turkish dataset (including four subjects with co-existent AS and FMF, three B51-positive and homozygote for rs61752717 ‘C’ allele, one HLA-B51-negative homozygote for rs61752717 ‘T’ allele). (DOCX)

S3 Table Genotype counts and (%) of rs61752717 in *HLA-B51*-positive and *HLA-B51*-negative cohorts in Iranian dataset. (DOCX)

S4 Table Genotype counts and (%) of rs61752717 in *HLA-B51*-positive and *HLA-B51*-negative cohorts in combined dataset (including four subjects with co-existent AS and FMF, three B51-positive and homozygote for rs61752717 ‘C’ allele, one HLA-B51-negative homozygote for rs61752717 ‘T’ allele). (DOCX)

S1 Fig Manhattan plot for chr1-22 (genotyped SNPs) in the Iranian cohort. (TIF)

S2 Fig Manhattan plot for chr1-22 (genotyped SNPs) in the Turkish cohort. (TIF)

S3 Fig Locus association plot of chromosome 2p15 and linkage disequilibrium with lead SNP rs13001372 in the Iranian cohort. (TIF)

S4 Fig Locus association plot of chromosome 5q15 and linkage disequilibrium with lead SNP rs27529 in the Iranian cohort. (TIF)

S5 Fig Locus association plot of chromosome 9q21.13 and linkage disequilibrium with lead SNP rs7874251 in the Iranian cohort. (TIF)

S6 Fig Locus association plot of chromosome 10q23.31 and linkage disequilibrium with lead SNP rs2031610 in the Iranian cohort. (TIF)

S7 Fig Locus association plot of chromosome 12p12.1 and linkage disequilibrium with lead SNP rs1488826 in the Iranian cohort. (TIF)

S8 Fig Locus association plot of chromosome 19p13.11 and linkage disequilibrium with lead SNP rs4808624 in the Iranian cohort. (TIF)

S9 Fig Locus association plot of chromosome 4p15.33 and linkage disequilibrium with lead SNP rs10000518 in the Turkish cohort. (TIF)

S10 Fig Locus association plot of chromosome 4p15.2 and linkage disequilibrium with lead SNP rs34939008 in the Turkish cohort. (TIF)

S11 Fig Locus association plot of chromosome 7p14 and linkage disequilibrium with lead SNP rs10280089 in the Turkish cohort. (TIF)

S12 Fig Locus association plot of chromosome 8p21.2 and linkage disequilibrium with lead SNP rs1013210 in the Turkish cohort. (TIF)

S13 Fig Locus association plot of chromosome 8p23.3 and linkage disequilibrium with lead SNP rs4876208 in the Turkish cohort. (TIF)

S14 Fig Locus association plot of chromosome 11q21 and linkage disequilibrium with lead SNP rs1271188 in the Turkish cohort. (TIF)

S15 Fig Locus association plot of chromosome 15q21.2 and linkage disequilibrium with lead SNP EXM1161045 in the Turkish cohort. (TIF)

S16 Fig Locus association plot of chromosome 15q26.3 and linkage disequilibrium with lead SNP rs8036083 in the Turkish cohort. (TIF)

S17 Fig Locus association plot of chromosome 15q23 and linkage disequilibrium with lead SNP rs11639037 in the Turkish cohort. (TIF)

S18 Fig Locus association plot of chromosome 16p13.3 and linkage disequilibrium with lead SNP rs61752717 in the Turkish cohort. (TIF)

S19 Fig Locus association plot of chromosome 18p11.21 and linkage disequilibrium with lead variant chr18:14723700:D in the Turkish cohort. (TIF)

S20 Fig Locus association plot of chromosome 21q22.3 and linkage disequilibrium with lead SNP rs235316 in the Turkish cohort. (TIF)

S21 Fig Plot of λ and λ_1000_ in Iranian and Turkish cohorts for non-MHC region. λ and λ_1000_ are coloured by red and blue, respectively. The x-axis and y-axis are the 1^st^ and corresponding λ and λ_1000_ values.
(TIF)

S22 Fig Plot of λ and λ_1000_ in the combined cohort for non-MHC region. λ and λ_1000_ are coloured by red and blue, respectively. The x-axis and y-axis are the 1^st^ and corresponding λ and λ_1000_ values.
(TIF)

S23 Fig Plot of principal component analysis (PCA) in the Iranian cohort. Healthy controls (HC) are in red while ankylosing spondylitis patients (AS) are in blue. The x-axis and y-axis are the 1^st^ and 2^nd^ principal component from the final PCA.
(TIF)

S24 Fig Plot of principal component analysis (PCA) in the Turkish cohort. Healthy controls (HC) are in red while ankylosing spondylitis patients (AS) are in blue. The x-axis and y-axis are the 1^st^ and 2^nd^ principal component from the final PCA.
(TIF)

S25 Fig Plot of principal component analysis (PCA) in the combined cohort of Turkish and Iranian samples. Iranian samples are in red while Turkish samples are in blue. The ankylosing spondylitis (AS) and healthy control (HC) are indicated by solid dots and triangles, respectively. The x-axis and y-axis are the 1^st^ and 2^nd^ principal component from the final PCA.
(TIF)

S26 Fig Q-Q plot of the Iranian cohort. The λ_1000_ was 1.041 for the entire dataset and 1.022 excluding the MHC region. Red dashed lines indicate 95% CI of expected χ^2^ values, while magenta and blue points are the observed χ^2^ values of the entire dataset and the dataset excluding the MHC region, respectively.
(TIF)

S27 Fig Q-Q plot of Turkish cohort, with first five PCA components as covariates. The λ_1000_ was 1.051 for overall dataset and 1.024 excluding MHC region. Red dashed lines indicate 95% CI of expected χ^2^ values, while magenta and blue points are the overserved χ^2^ values of the entire dataset and dataset excluding the MHC region, respectively.
(TIF)

S28 Fig Q-Q plot of the combined cohort (with first four PCA components as covariates). The λ_1000_ was 1.086 for the entire dataset and 1.021 excluding the MHC region. Red dashed lines indicate 95% CI of expected χ^2^ values, while magenta and black points are the overserved χ^2^ values of the entire dataset and dataset excluding the MHC region, respectively.
(TIF)

## References

[1] Brown MA, Kennedy LG, MacGregor AJ, Darke C, Duncan E, Shatford JL, et al Susceptibility to ankylosing spondylitis in twins: the role of genes, HLA, and the environment. Arthritis Rheum. 1997;40(10):1823–8. . 9336417 10.1002/art.1780401015

[2] Pedersen OB, Svendsen AJ, Ejstrup L, Skytthe A, Harris JR, Junker P. Ankylosing spondylitis in Danish and Norwegian twins: occurrence and the relative importance of genetic vs. environmental effectors in disease causation. Scand J Rheumatol. 2008;37(2):120–6. Epub 2008/04/17. 10.1080/03009740701824613 . 18415769

[3] Ellinghaus D, Jostins L, Spain SL, Cortes A, Bethune J, Han B, et al Analysis of five chronic inflammatory diseases identifies 27 new associations and highlights disease-specific patterns at shared loci. Nat Genet. 2016;48(5):510–8. 10.1038/ng.3528 . 26974007 PMC4848113

[4] Lehman TJ, Hanson V, Kornreich H, Peters RS, Schwabe AD. HLA-B27-negative sacroiliitis: a manifestation of familial Mediterranean fever in childhood. Pediatrics. 1978;61(3):423–6. . 643416 10.1542/peds.61.3.423

[5] Akar S, Soysal O, Balci A, Solmaz D, Gerdan V, Onen F, et al High prevalence of spondyloarthritis and ankylosing spondylitis among familial Mediterranean fever patients and their first-degree relatives: further evidence for the connection. Arthritis Res Ther. 2013;15(1):R21 10.1186/ar4154 23356447 PMC3672706

[6] Kasifoglu T, Calisir C, Cansu DU, Korkmaz C. The frequency of sacroiliitis in familial Mediterranean fever and the role of HLA-B27 and MEFV mutations in the development of sacroiliitis. Clin Rheumatol. 2009;28(1):41–6. 10.1007/s10067-008-0980-3 . 18795391

[7] Akkoc N, Gul A. Familial Mediterranean Fever and Seronegative Arthritis. Current rheumatology reports. 2011;13(5):388–94. 10.1007/s11926-011-0191-9 21695514

[8] Langevitz P, Livneh A, Zemer D, Shemer J, Pras M. Seronegative spondyloarthropathy in familial Mediterranean fever. Semin Arthritis Rheum. 1997;27(2):67–72. . 9355205 10.1016/s0049-0172(97)80007-8

[9] Balaban B, Yasar E, Ozgul A, Dincer K, Kalyon TA. Sacroiliitis in familial Mediterranean fever and seronegative spondyloarthropathy: importance of differential diagnosis. Rheumatol Int. 2005;25(8):641–4. 10.1007/s00296-004-0578-2 . 15711787

[10] Durmus D, Alayli G, Cengiz K, Yigit S, Canturk F, Bagci H. Clinical significance of MEFV mutations in ankylosing spondylitis. Joint Bone Spine. 2009;76(3):260–4. 10.1016/j.jbspin.2008.09.011 . 19119044

[11] Cosan F, Ustek D, Oku B, Duymaz-Tozkir J, Cakiris A, Abaci N, et al Association of familial Mediterranean fever-related MEFV variations with ankylosing spondylitis. Arthritis Rheum. 2010;62(11):3232–6. 10.1002/art.27683 . 20669279

[12] Akkoc N, Sari I, Akar S, Binicier O, Thomas MG, Weale ME, et al Increased prevalence of M694V in patients with ankylosing spondylitis: additional evidence for a link with familial mediterranean fever. Arthritis Rheum. 2010;62(10):3059–63. 10.1002/art.27598 . 20533539

[13] Akkoc N, Gul A. Comment on the article by Durmus et al. "Clinical significance of MEFV mutations in ankylosing spondylitis". Joint Bone Spine. 2010;77(3):281 10.1016/j.jbspin.2009.12.008 . 20356777

[14] Yigit S, Inanir A, Karakus N, Kesici E, Bozkurt N. Common Mediterranean fever (MEFV) gene mutations associated with ankylosing spondylitis in Turkish population. Dis Markers. 2012;33(3):113–8. 10.3233/DMA-2012-0911 22960328 PMC3810788

[15] Touitou I, Magne X, Molinari N, Navarro A, Quellec AL, Picco P, et al MEFV mutations in Behcet’s disease. Hum Mutat. 2000;16(3):271–2. 10.1002/1098-1004(200009)16:3<271::AID-HUMU16>3.0.CO;2-A . 10980540

[16] Wu Z, Zhang S, Li J, Chen S, Li P, Sun F, et al Association between MEFV Mutations M694V and M680I and Behcet’s Disease: A Meta-Analysis. PLoS One. 2015;10(7):e0132704 10.1371/journal.pone.0132704 26176758 PMC4503748

[17] Atagunduz P, Ergun T, Direskeneli H. MEFV mutations are increased in Behcet’s disease (BD) and are associated with vascular involvement. Clin Exp Rheumatol. 2003;21(4 Suppl 30):S35–7. . 14727457

[18] Kirino Y, Zhou Q, Ishigatsubo Y, Mizuki N, Tugal-Tutkun I, Seyahi E, et al Targeted resequencing implicates the familial Mediterranean fever gene MEFV and the toll-like receptor 4 gene TLR4 in Behcet disease. Proc Natl Acad Sci U S A. 2013;110(20):8134–9. 10.1073/pnas.1306352110 23633568 PMC3657824

[19] Ait Badi MA, Zyani M, Kaddouri S, Niamane R, Hda A, Algayres JP. Skeletal manifestations in Behcet’s disease. A report of 79 cases. Rev Med Interne. 2008;29(4):277–82. 10.1016/j.revmed.2007.09.031 . 18289738

[20] Ohno S, Asanuma T, Sugiura S, Wakisaka A, Aizawa M, Itakura K. HLA-Bw51 and Behcet’s disease. JAMA. 1978;240(6):529 . 671660

[21] Cortes A, Pulit SL, Leo PJ, Pointon JJ, Robinson PC, Weisman MH, et al Major histocompatibility complex associations of ankylosing spondylitis are complex and involve further epistasis with ERAP1. Nat Commun. 2015;6:7146 10.1038/ncomms8146 25994336 PMC4443427

[22] Haibel H, Rudwaleit M, Listing J, Sieper J. Open label trial of anakinra in active ankylosing spondylitis over 24 weeks. Ann Rheum Dis. 2005;64(2):296–8. 10.1136/ard.2004.023176 15208175 PMC1755331

[23] Manukyan GP, Ghazaryan KA, Ktsoyan Zh A, Tatyan MV, Khachatryan ZA, Hakobyan GS, et al Cytokine profile of Armenian patients with Familial Mediterranean fever. Clin Biochem. 2008;41(10–11):920–2. 10.1016/j.clinbiochem.2008.03.017 . 18440310

[24] Ovadia A, Livneh A, Feld O, Ben-Zvi I, Kukuy E, Kivity S, et al T helper 17 polarization in familial Mediterranean fever. Genes Immun. 2013;14(4):212–6. 10.1038/gene.2013.6 . 23466494

[25] Robinson WP, van der Linden SM, Khan MA, Rentsch HU, Cats A, Russell A, et al HLA-Bw60 increases susceptibility to ankylosing spondylitis in HLA-B27+ patients. Arthritis Rheum. 1989;32(9):1135–41. . 2789045 10.1002/anr.1780320912

[26] Brown MA, Pile KD, Kennedy LG, Calin A, Darke C, Bell J, et al HLA class I associations of ankylosing spondylitis in the white population in the United Kingdom. Ann Rheum Dis. 1996;55(4):268–70. . 8733445 10.1136/ard.55.4.268 PMC1010149

[27] Chae JJ, Komarow HD, Cheng J, Wood G, Raben N, Liu PP, et al Targeted Disruption of Pyrin, the FMF Protein, Causes Heightened Sensitivity to Endotoxin and a Defect in Macrophage Apoptosis. Molecular Cell. 2003;11(3):591–604. 10.1016/s1097-2765(03)00056-x 12667444

[28] Chae JJ, Wood G, Masters SL, Richard K, Park G, Smith BJ, et al The B30.2 domain of pyrin, the familial Mediterranean fever protein, interacts directly with caspase-1 to modulate IL-1beta production. Proc Natl Acad Sci U S A. 2006;103(26):9982–7. 10.1073/pnas.0602081103 16785446 PMC1479864

[29] Chae JJ, Cho YH, Lee GS, Cheng J, Liu PP, Feigenbaum L, et al Gain-of-function Pyrin mutations induce NLRP3 protein-independent interleukin-1beta activation and severe autoinflammation in mice. Immunity. 2011;34(5):755–68. 10.1016/j.immuni.2011.02.020 21600797 PMC3129608

[30] Kimura T, Jain A, Choi SW, Mandell MA, Schroder K, Johansen T, et al TRIM-mediated precision autophagy targets cytoplasmic regulators of innate immunity. J Cell Biol. 2015;210(6):973–89. 10.1083/jcb.201503023 26347139 PMC4576868

[31] Ambarus C, Yeremenko N, Tak PP, Baeten D. Pathogenesis of spondyloarthritis: autoimmune or autoinflammatory? Curr Opin Rheumatol. 2012;24(4):351–8. 10.1097/BOR.0b013e3283534df4 . 22488076

[32] Ciccia F, Haroon N. Autophagy in the pathogenesis of ankylosing spondylitis. Clin Rheumatol. 2016;35(6):1433–6. 10.1007/s10067-016-3262-5 . 27075464

[33] Park MC, Kim HW, Lee SW, Song JJ, Park YB. Defective autophagy activity and its association with spinal damage in patients with ankylosing spondylitis. Joint Bone Spine. 2017;84(5):583–7. 10.1016/j.jbspin.2016.09.005 . 27825566

[34] Ciccia F, Guggino G, Rizzo A, Saieva L, Peralta S, Giardina A, et al Type 3 innate lymphoid cells producing IL-17 and IL-22 are expanded in the gut, in the peripheral blood, synovial fluid and bone marrow of patients with ankylosing spondylitis. Ann Rheum Dis. 2015;74(9):1739–47. 10.1136/annrheumdis-2014-206323 . 25902790

[35] Gracey E, Qaiyum Z, Almaghlouth I, Lawson D, Karki S, Avvaru N, et al IL-7 primes IL-17 in mucosal-associated invariant T (MAIT) cells, which contribute to the Th17-axis in ankylosing spondylitis. Ann Rheum Dis. 2016;75(12):2124–32. 10.1136/annrheumdis-2015-208902 . 27165176

[36] Kenna TJ, Davidson SI, Duan R, Bradbury LA, McFarlane J, Smith M, et al Enrichment of circulating interleukin-17-secreting interleukin-23 receptor-positive gamma/delta T cells in patients with active ankylosing spondylitis. Arthritis Rheum. 2012;64(5):1420–9. 10.1002/art.33507 . 22144400

[37] International Genetics of Ankylosing Spondylitis C, Cortes A, Hadler J, Pointon JP, Robinson PC, Karaderi T, et al Identification of multiple risk variants for ankylosing spondylitis through high-density genotyping of immune-related loci. Nat Genet. 2013;45(7):730–8. 10.1038/ng.2667 23749187 PMC3757343

[38] Toussirot E, Lafforgue P, Boucraut J, Despieds P, Schiano A, Bernard D, et al Serum levels of interleukin 1-beta, tumor necrosis factor-alpha, soluble interleukin 2 receptor and soluble CD8 in seronegative spondylarthropathies. Rheumatol Int. 1994;13(5):175–80. . 8202660 10.1007/BF00390264

[39] Gratacos J, Collado A, Filella X, Sanmarti R, Canete J, Llena J, et al Serum cytokines (IL-6, TNF-alpha, IL-1 beta and IFN-gamma) in ankylosing spondylitis: a close correlation between serum IL-6 and disease activity and severity. Br J Rheumatol. 1994;33(10):927–31. . 7921752 10.1093/rheumatology/33.10.927

[40] Bal A, Unlu E, Bahar G, Aydog E, Eksioglu E, Yorgancioglu R. Comparison of serum IL-1 beta, sIL-2R, IL-6, and TNF-alpha levels with disease activity parameters in ankylosing spondylitis. Clin Rheumatol. 2007;26(2):211–5. 10.1007/s10067-006-0283-5 . 16583185

[41] Tan AL, Marzo-Ortega H, O'Connor P, Fraser A, Emery P, McGonagle D. Efficacy of anakinra in active ankylosing spondylitis: a clinical and magnetic resonance imaging study. Ann Rheum Dis. 2004;63(9):1041–5. 10.1136/ard.2004.020800 15066864 PMC1755137

[42] Estublier C, Stankovic Stojanovic K, Bergerot JF, Broussolle C, Seve P. Myositis in a patient with familial Mediterranean fever and spondyloarthritis successfully treated with anakinra. Joint Bone Spine. 2013;80(6):645–9. 10.1016/j.jbspin.2013.03.004 . 23928237

[43] Georgin-Lavialle S, Stankovic Stojanovic K, Bachmeyer C, Sellam J, Abbara S, Awad F, et al Spondyloarthritis associated with familial Mediterranean fever: successful treatment with anakinra. Rheumatology (Oxford). 2017;56(1):167–9. 10.1093/rheumatology/kew290 . 27576367

[44] Varan O, Kucuk H, Tufan A. Anakinra for the treatment of familial Mediterranean fever-associated spondyloarthritis. Scand J Rheumatol. 2016;45(3):252–3. 10.3109/03009742.2015.1127413 . 26948937

[45] Costello ME, Ciccia F, Willner D, Warrington N, Robinson PC, Gardiner B, et al Intestinal dysbiosis in ankylosing spondylitis. Arthritis & rheumatology. 2014;67(3):686–91. 10.1002/art.38967 . 25417597

[46] Akyuz F, Besisik F, Ustek D, Ekmekci C, Uyar A, Pinarbasi B, et al Association of the MEFV gene variations with inflammatory bowel disease in Turkey. J Clin Gastroenterol. 2013;47(3):e23–7. 10.1097/MCG.0b013e3182597992 . 22810105

[47] Uslu N, Yuce A, Demir H, Saltik-Temizel IN, Usta Y, Yilmaz E, et al The association of inflammatory bowel disease and Mediterranean fever gene (MEFV) mutations in Turkish children. Dig Dis Sci. 2010;55(12):3488–94. 10.1007/s10620-010-1178-5 . 20306331

[48] Khachatryan ZA, Ktsoyan ZA, Manukyan GP, Kelly D, Ghazaryan KA, Aminov RI. Predominant role of host genetics in controlling the composition of gut microbiota. PLoS One. 2008;3(8):e3064 10.1371/journal.pone.0003064 18725973 PMC2516932

[49] Delaneau O, Marchini J, Zagury JF. A linear complexity phasing method for thousands of genomes. Nat Methods. 2011;9(2):179–81. 10.1038/nmeth.1785 . 22138821

[50] Howie BN, Donnelly P, Marchini J. A flexible and accurate genotype imputation method for the next generation of genome-wide association studies. PLoS Genet. 2009;5(6):e1000529 Epub 2009/06/23. 10.1371/journal.pgen.1000529 19543373 PMC2689936

[51] Jia X, Han B, Onengut-Gumuscu S, Chen WM, Concannon PJ, Rich SS, et al Imputing amino acid polymorphisms in human leukocyte antigens. PLoS One. 2013;8(6):e64683 10.1371/journal.pone.0064683 23762245 PMC3675122

[52] Chang CC, Chow CC, Tellier LC, Vattikuti S, Purcell SM, Lee JJ. Second-generation PLINK: rising to the challenge of larger and richer datasets. Gigascience. 2015;4(1):1–16. 10.1186/s13742-015-0047-8 25722852 PMC4342193

[53] Willer CJ, Li Y, Abecasis GR. METAL: fast and efficient meta-analysis of genomewide association scans. Bioinformatics. 2010;26(17):2190–1. 10.1093/bioinformatics/btq340 20616382 PMC2922887

