# Supplementary material for: Genome-wide association study in Turkish and Iranian populations identify rare familial Mediterranean fever gene (MEFV) polymorphisms associated with ankylosing spondylitis
Source: PLoS Genet. 2019 Apr 4;15(4):e1008038. doi: 10.1371/journal.pgen.1008038 (PMC6467421; doi:10.1371/journal.pgen.1008038)
Supplement: S1 Table — (DOCX) [file pgen.1008038.s001.docx]

|  |  | SNP | *P*-value | OR (95%CI) | position | AA mutation | DNA mutation |
| --- | --- | --- | --- | --- | --- | --- | --- |
| Turkish | Coding | rs61752717 | 7.63×10^−12^ | 5.34 (3.31, 8.62) | 3293407 | M694V | 2080A>G |
|  |  | rs224224 | 0.014 | 1.18 (1.04,1.35) | 3304654 | G138G | 414A->G |
|  |  | rs224223 | 0.015 | 1.18 (1.03, 1.35) | 3304573 | A165A | 495C->A |
|  |  | rs224225 | 0.016 | 1.18 (1.03, 1.35) | 3304762 | D102D | 305T->C |
|  | Non-coding | rs224216 | 0.011 | 1.19 (1.04, 1.36) | 3301621 |  | T->C |
|  |  | rs224219 | 0.013 | 1.19 (1.04, 1.35) | 3302313 |  | C->T |
|  |  | rs224226 | 0.01383 | 1.18 (1.04, 1.35) | 3305732 |  | A->G |
|  |  | rs224218 | 0.017 | 1.18 (1.03, 1.34) | 3301897 |  | T->C |
|  |  | rs224217 | 0.019 | 1.172 (1.03, 1.34) | 3301757 |  | C->T |
|  |  | rs224215 | 0.020 | 1.19 (1.03, 1.37) | 3301360 |  | T->C |
|  |  | rs224221 | 0.034 | 1.16 (1.01, 1.32) | 3303531 |  | T->A |
| Iranian | Coding | EXM1210719/  rs11466023 | 0.016 | 0.34 (0.14, 0.813) | 3299586 | P369S | 1105C>T |
|  |  | EXM1210711/  rs11466024 | 0.030 | 0.37 (0.15, 0.91) | 3299468 | R408Q | 1223G>A |
|  |  | rs61752717 | 0.042 | 2.85 (1.04, 7.83) | 3293407 | M694V | 2080A>G |

**S1 Table.** AS-associated variations in *MEFV* in Turkish and Iranian case-control cohorts separately.
