# Supplementary material for: Genome-wide association study in Turkish and Iranian populations identify rare familial Mediterranean fever gene (MEFV) polymorphisms associated with ankylosing spondylitis
Source: PLoS Genet. 2019 Apr 4;15(4):e1008038. doi: 10.1371/journal.pgen.1008038 (PMC6467421; doi:10.1371/journal.pgen.1008038)
Supplement: S2 Table — (DOCX) [file pgen.1008038.s002.docx]

**S2 Table.** Genotype counts and (%) of rs61752717 in *HLA-B51*-positive and *HLA-B51*-negative cohorts in Turkish dataset (including four subjects with co-existent AS and FMF, three B51-positive and homozygote for rs61752717 ‘C’ allele, one HLA-B51-negative homozygote for rs61752717 ‘T’ allele).

| rs61752717 | Case | | Control | | Total |
| --- | --- | --- | --- | --- | --- |
|  | B51+ | B51− | B51+ | B51− |  |
| CC (+/+) | 2 | 5 (0.7) | 0 (0.0) | 0 (0.0) | 10 |
| CT (+/−) | 15 (8.5) | 76 (10.3) | 5 (2.5) | 14 (2.0) | 110 |
| TT (−/−) | 156 (88.6) | 658 (89.0) | 198 (97.5) | 683 (98.0) | 1695 |
| CC or CT (+/+ or +/−) | 20 (11.4) | 81 (11.0) | 5 (2.5) | 14 (2.0) | 120 |
| Sum | 176 | 739 | 203 | 697 | 1815 |

rs61752717 genotype counts differ marginally between *HLA-B51*-positive and –negative cases (*P=*0.038). Comparing allele frequencies between *HLA-B51*-positive and –negative controls, no difference was observed (CC genotype frequency comparison not possible due to zero counts of CC genotypes; *P=*0.69). The association of rs61752717 in HLA-B51-negative cases was higher (OR=5.88, 95% CI=3.31-10.42, *P*=1.36×10^−9^ or OR=5.88, 95% CI=3.317- 10.43, *P*=1.33×10^−9^ if excluding four cases with co-existent FMF and AS) than in HLA-B51-positive cases (OR=4.82, 95% CI=1.72-11.66, *P*=2.11×10^−3^ or OR=4.24, 95% CI=1.58- 11.42, *P*=4.22×10^−3^ if excluding four cases with co-existent FMF and AS). The rs61752717 risk allele carriage was higher in the *HLA-B51* negative cohort than in the positive cohort (OR = 3.39, 95% CI 1.42-7.47, *P*=0.003)
