# Supplementary material for: Genome-wide association study in Turkish and Iranian populations identify rare familial Mediterranean fever gene (MEFV) polymorphisms associated with ankylosing spondylitis
Source: PLoS Genet. 2019 Apr 4;15(4):e1008038. doi: 10.1371/journal.pgen.1008038 (PMC6467421; doi:10.1371/journal.pgen.1008038)
Supplement: S3 Table — (DOCX) [file pgen.1008038.s003.docx]

**S3 Table.** Genotype counts and (%) of rs61752717 in *HLA-B51*-positive and *HLA-B51*-negative cohorts in Iranian dataset

| rs61752717 | Case | | Control | | Total |
| --- | --- | --- | --- | --- | --- |
|  | B51+ | B51− | B51+ | B51− |  |
| CC (+/+) | 1 (1.0) | 1 (0.3) | 0 (0) | 0 (0) | 2 |
| CT (+/−) | 1 (1.0) | 5 (1.6) | 0 (0) | 5 (0.9) | 11 |
| TT (−/−) | 94 (97.9) | 316 (98.1) | 179 (100) | 566 (99.1) | 1155 |
| CC or CT (+/+ or +/−) | 2 (2.1) | 6 (1.9) | 0 (0) | 5 (99.1) | 13 |
| Sum | 96 | 322 | 179 | 571 | 1168 |

No significant difference was observed in risk allele carriage between *HLA-B51*-negative cases and positive cases (OR = 0.68, 95% CI 0.15-4.10, *P* = 0.70)
