# Supplementary material for: Genome-wide association study in Turkish and Iranian populations identify rare familial Mediterranean fever gene (MEFV) polymorphisms associated with ankylosing spondylitis
Source: PLoS Genet. 2019 Apr 4;15(4):e1008038. doi: 10.1371/journal.pgen.1008038 (PMC6467421; doi:10.1371/journal.pgen.1008038)
Supplement: S4 Table — (DOCX) [file pgen.1008038.s004.docx]

**S4 Table.** Genotype counts and (%) of rs61752717 in *HLA-B51*-positive and *HLA-B51*-negative cohorts in combined dataset (including four subjects with co-existent AS and FMF, three B51-positive and homozygote for rs61752717 ‘C’ allele, one HLA-B51-negative homozygote for rs61752717 ‘T’ allele).

| rs61752717 | Case | | Control | | Total |
| --- | --- | --- | --- | --- | --- |
|  | B51+ | B51− | B51+ | B51− |  |
| CC (+/+) | 6 (2.2) | 6 (0.6) | 0 (0) | 0 (0) | 12 |
| CT (+/−) | 16 (5.9) | 81 (7.8) | 5 (1.3) | 19 (1.5) | 121 |
| TT (−/−) | 250 (91.9) | 947 (91.6) | 377 (98.7) | 1249 (98.5) | 2823 |
| CC or CT (+/+ or +/−) | 22 (8.1) | 87 (8.4) | 5 (1.3) | 19 (1.5) | 133 |
| Sum | 272 | 1034 | 382 | 1268 | 2956 |

No significant difference is observed in risk allele carriage between *HLA-B51*-negative cases and positive cases (OR = 0.87, 95% CI = 0.56-1.39, *P* = 0.49 or OR = 1.08, 95% CI 0.66-1.83, *P* = 0.81 if excluding four cases with co-existent FMF and AS)
