# Supplementary figures and images for: Genome-wide association study in Turkish and Iranian populations identify rare familial Mediterranean fever gene (MEFV) polymorphisms associated with ankylosing spondylitis
Source: PLoS Genet. 2019 Apr 4;15(4):e1008038. doi: 10.1371/journal.pgen.1008038 (PMC6467421; doi:10.1371/journal.pgen.1008038)

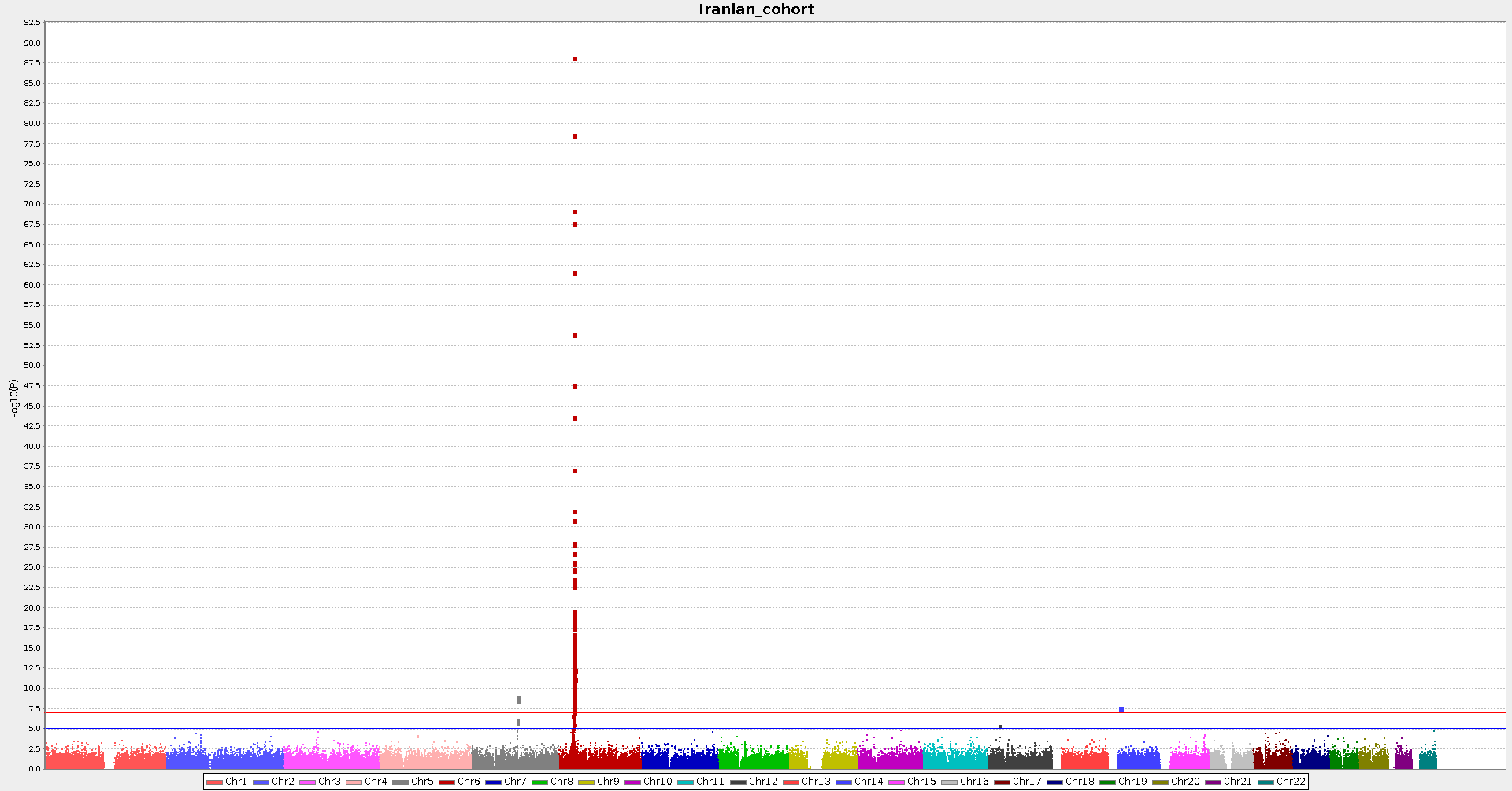

Supplement: S1 Fig — (TIF) [file pgen.1008038.s005.tif]

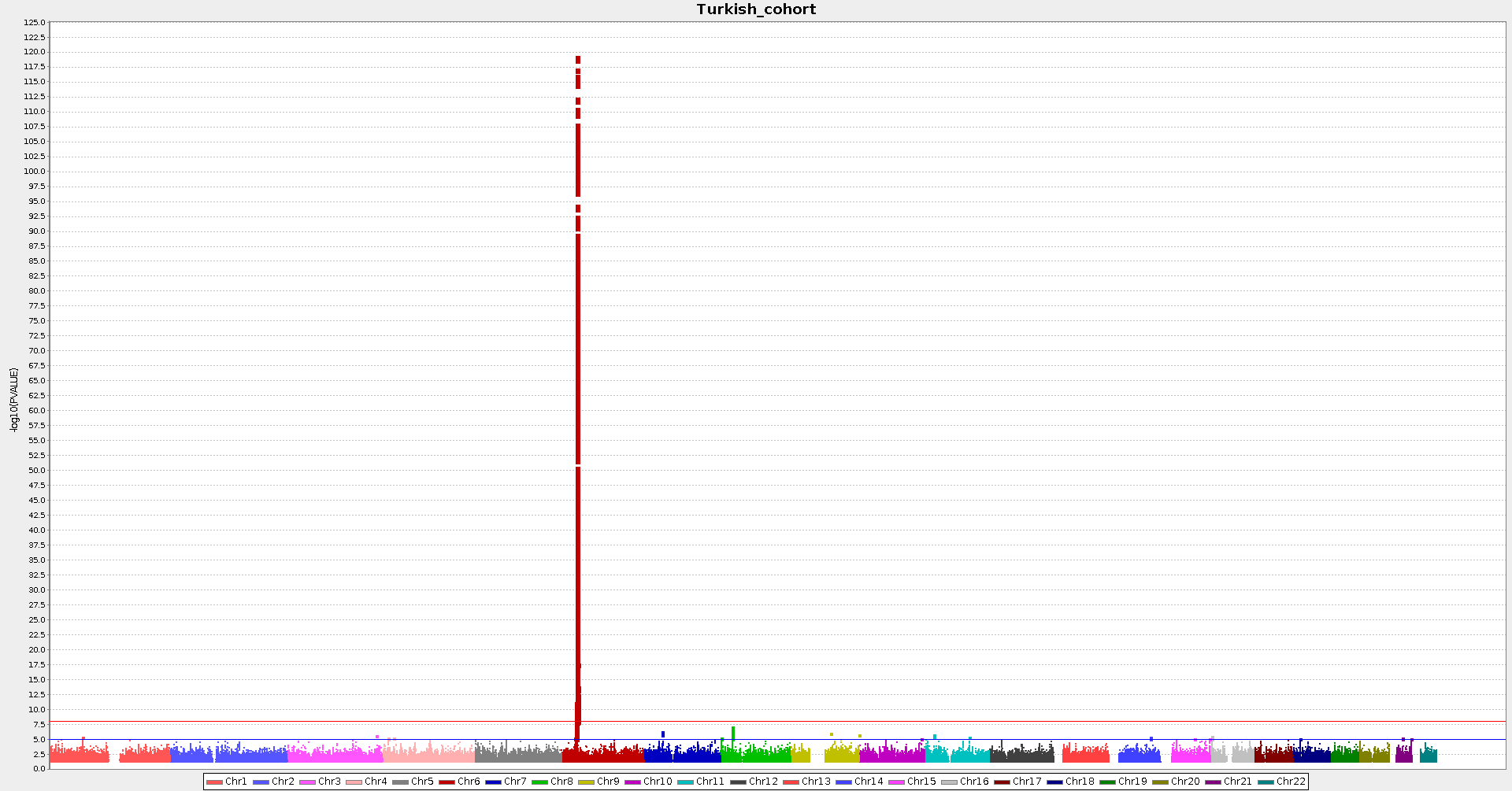

Supplement: S2 Fig — (TIF) [file pgen.1008038.s006.tif]

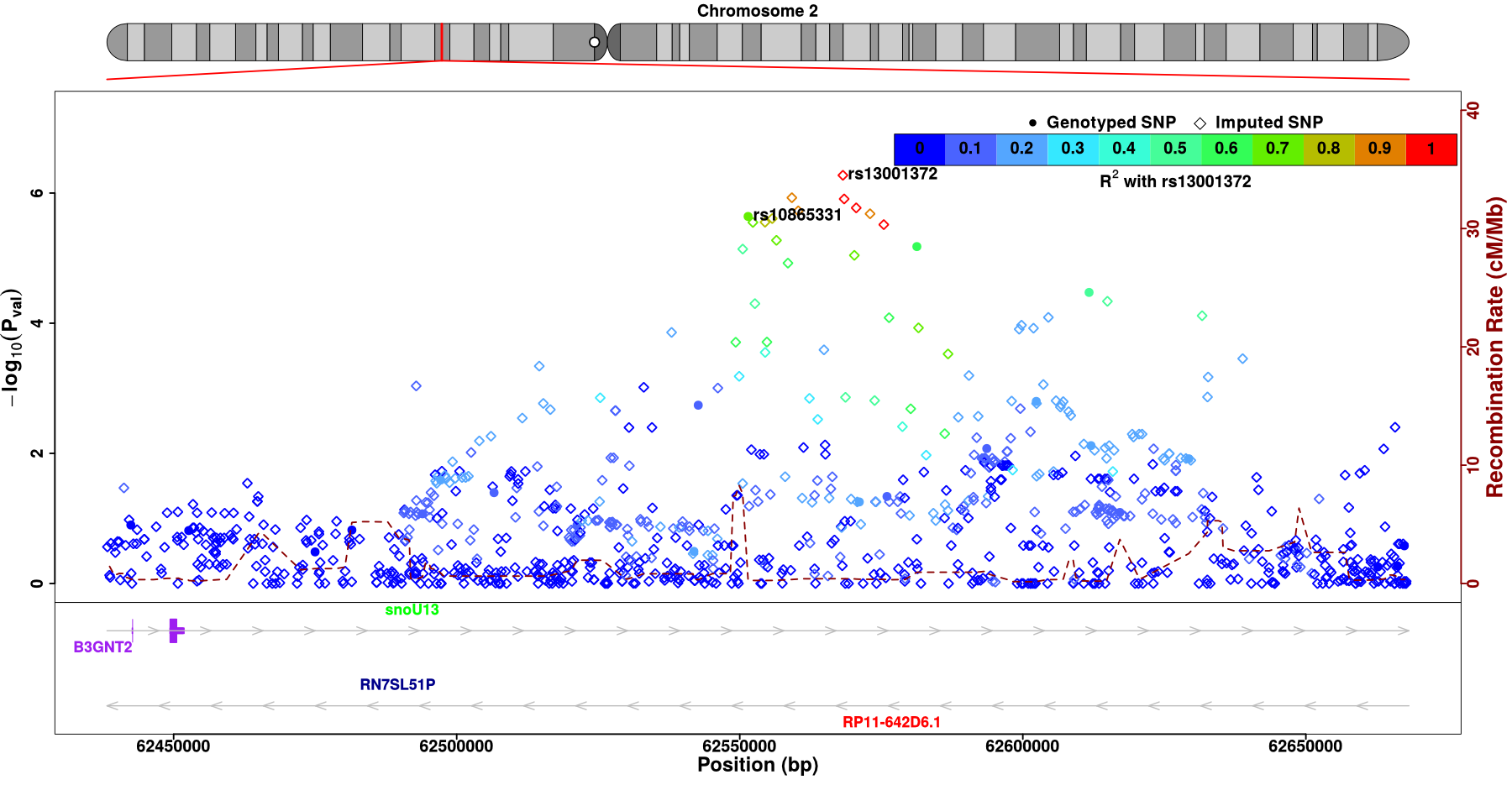

Supplement: S3 Fig — (TIF) [file pgen.1008038.s007.tif]

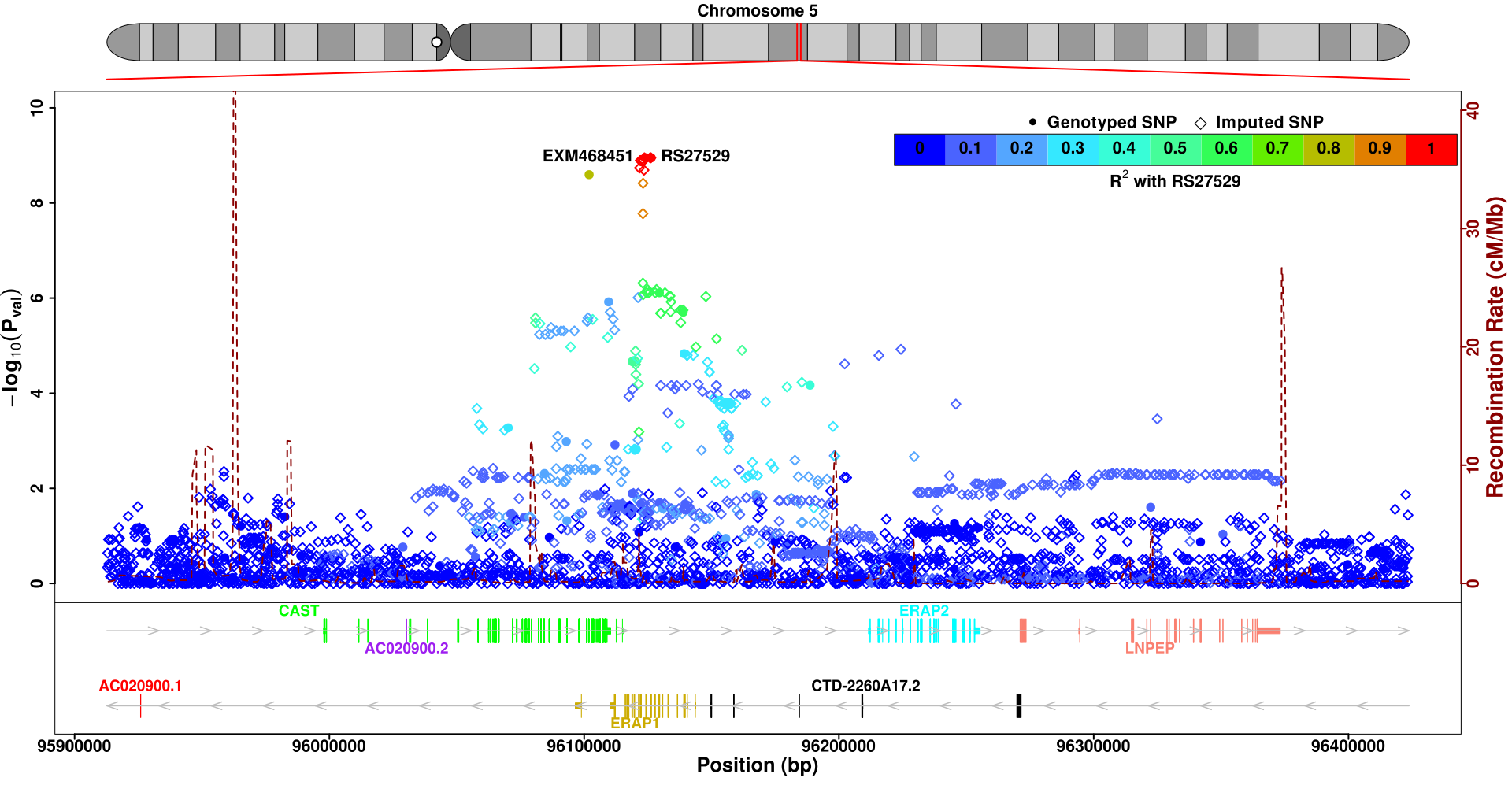

Supplement: S4 Fig — (TIF) [file pgen.1008038.s008.tif]

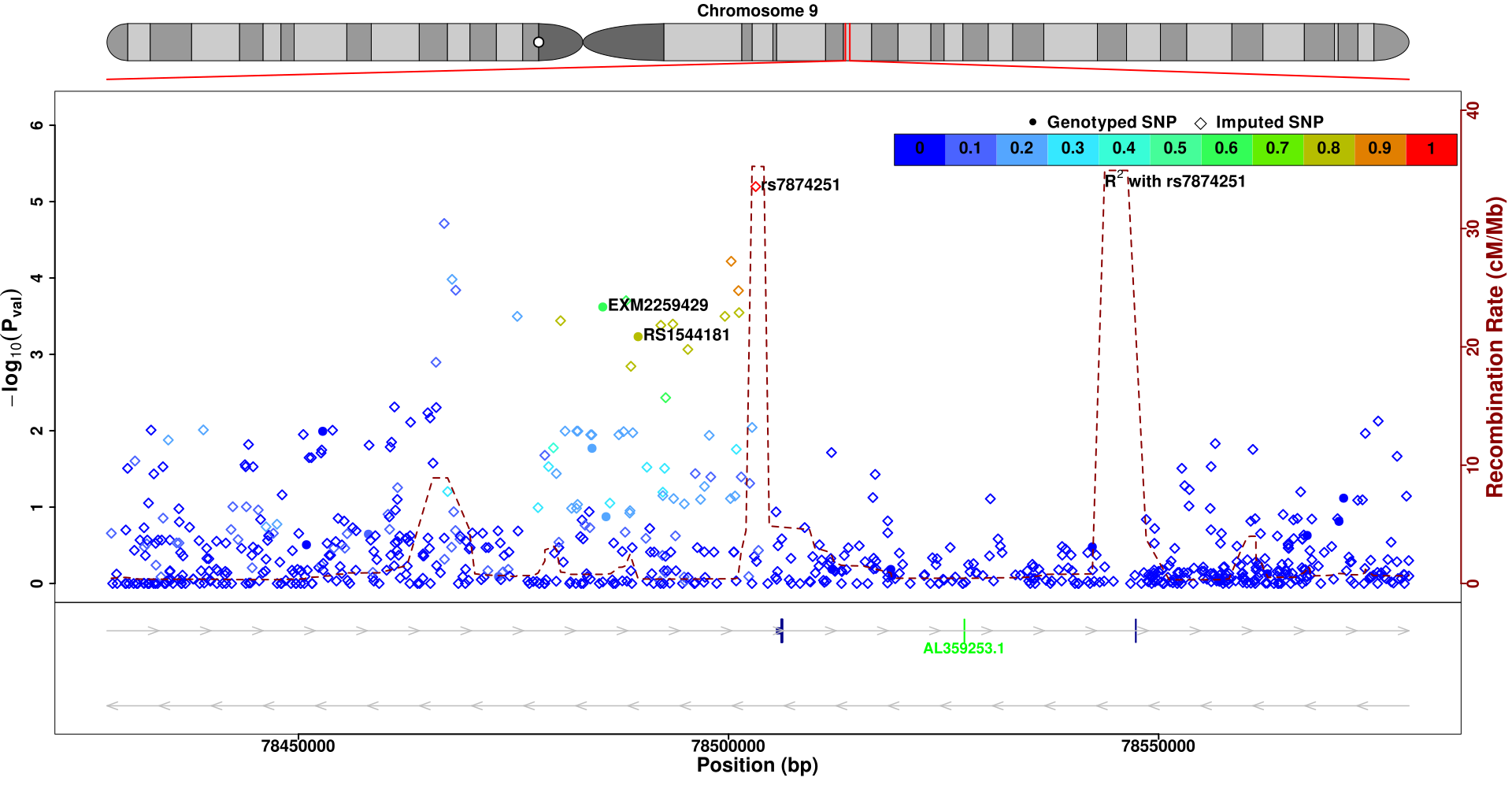

Supplement: S5 Fig — (TIF) [file pgen.1008038.s009.tif]

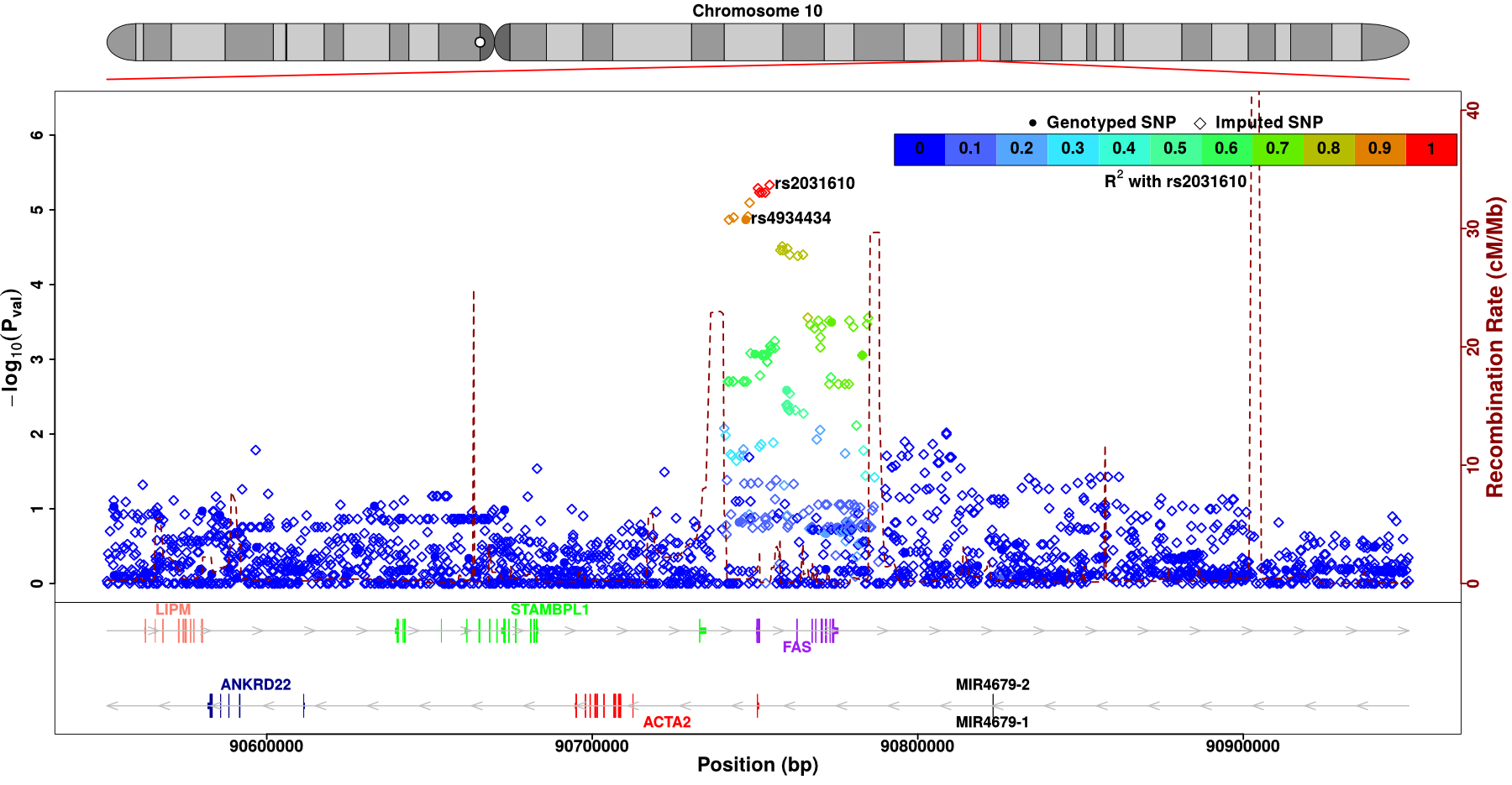

Supplement: S6 Fig — (TIF) [file pgen.1008038.s010.tif]

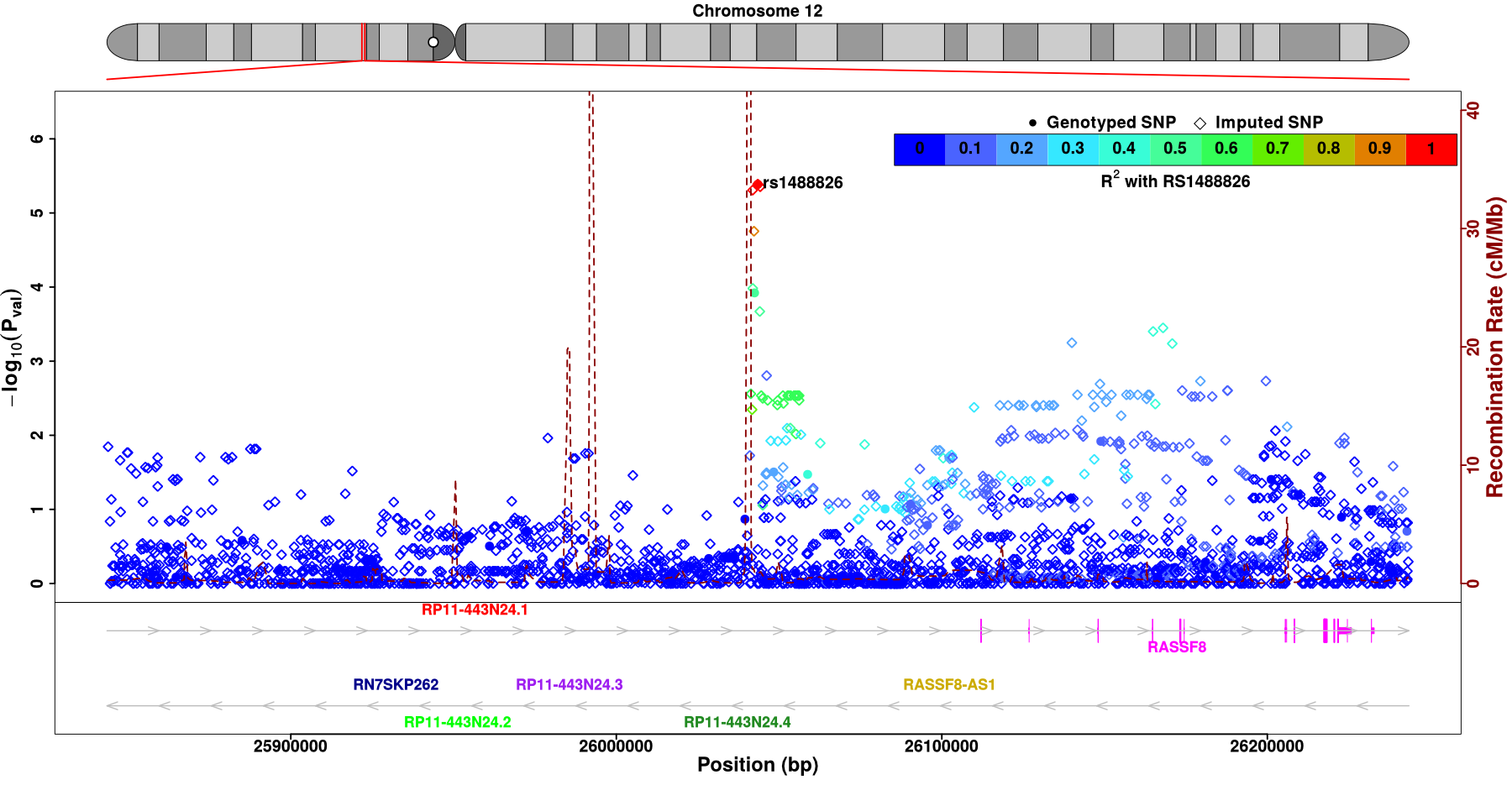

Supplement: S7 Fig — (TIF) [file pgen.1008038.s011.tif]

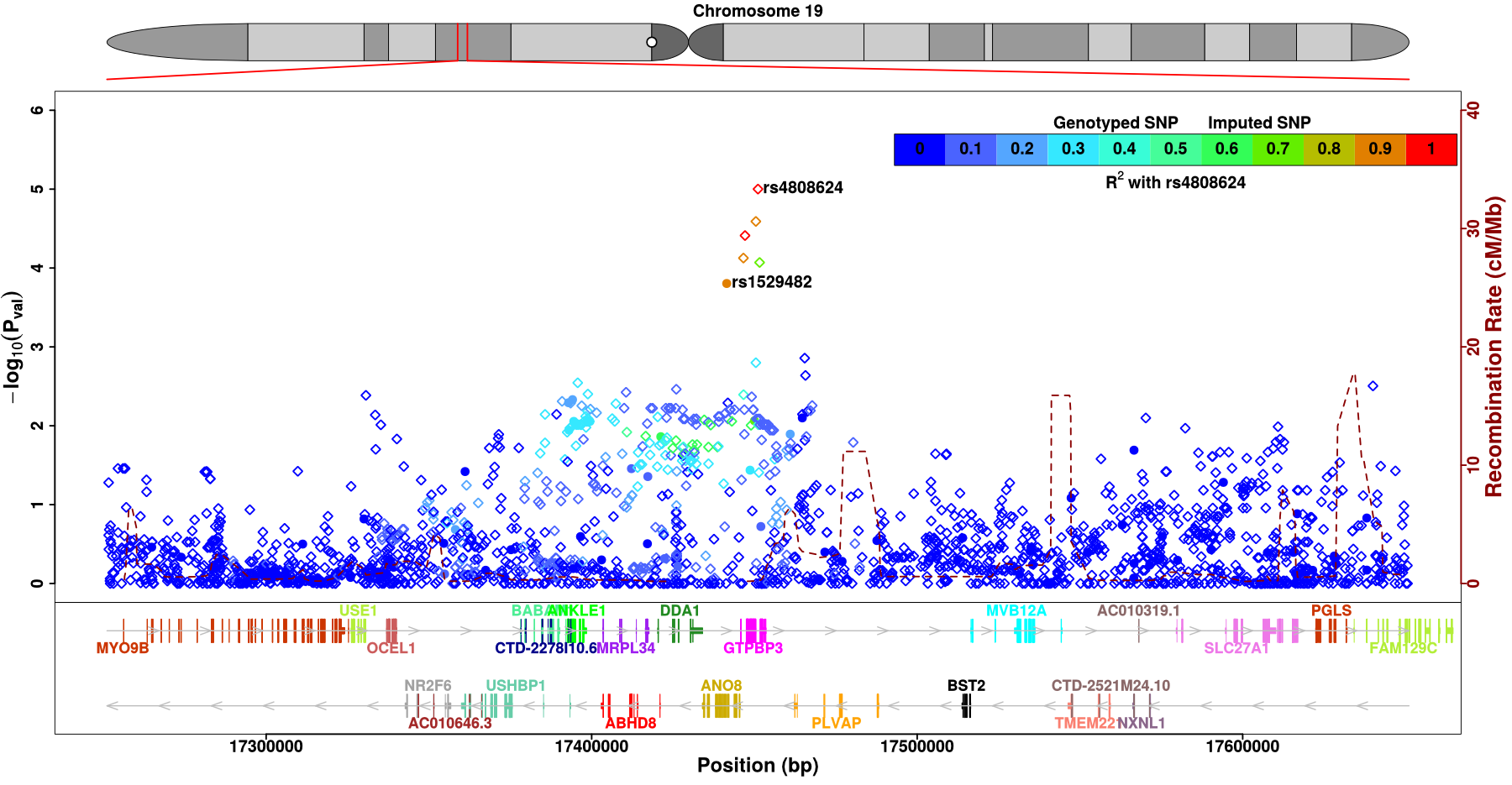

Supplement: S8 Fig — (TIF) [file pgen.1008038.s012.tif]

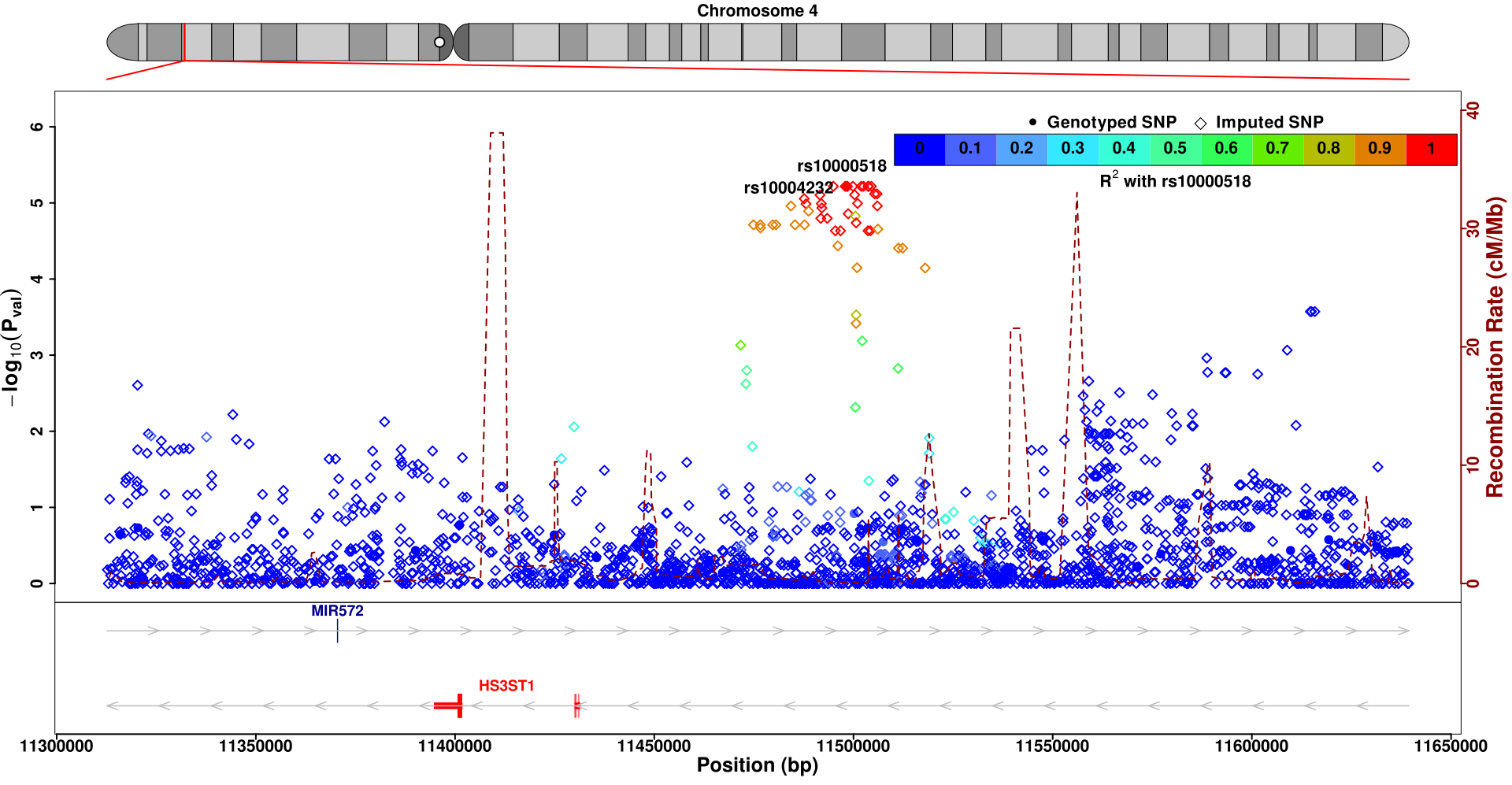

Supplement: S9 Fig — (TIF) [file pgen.1008038.s013.tif]

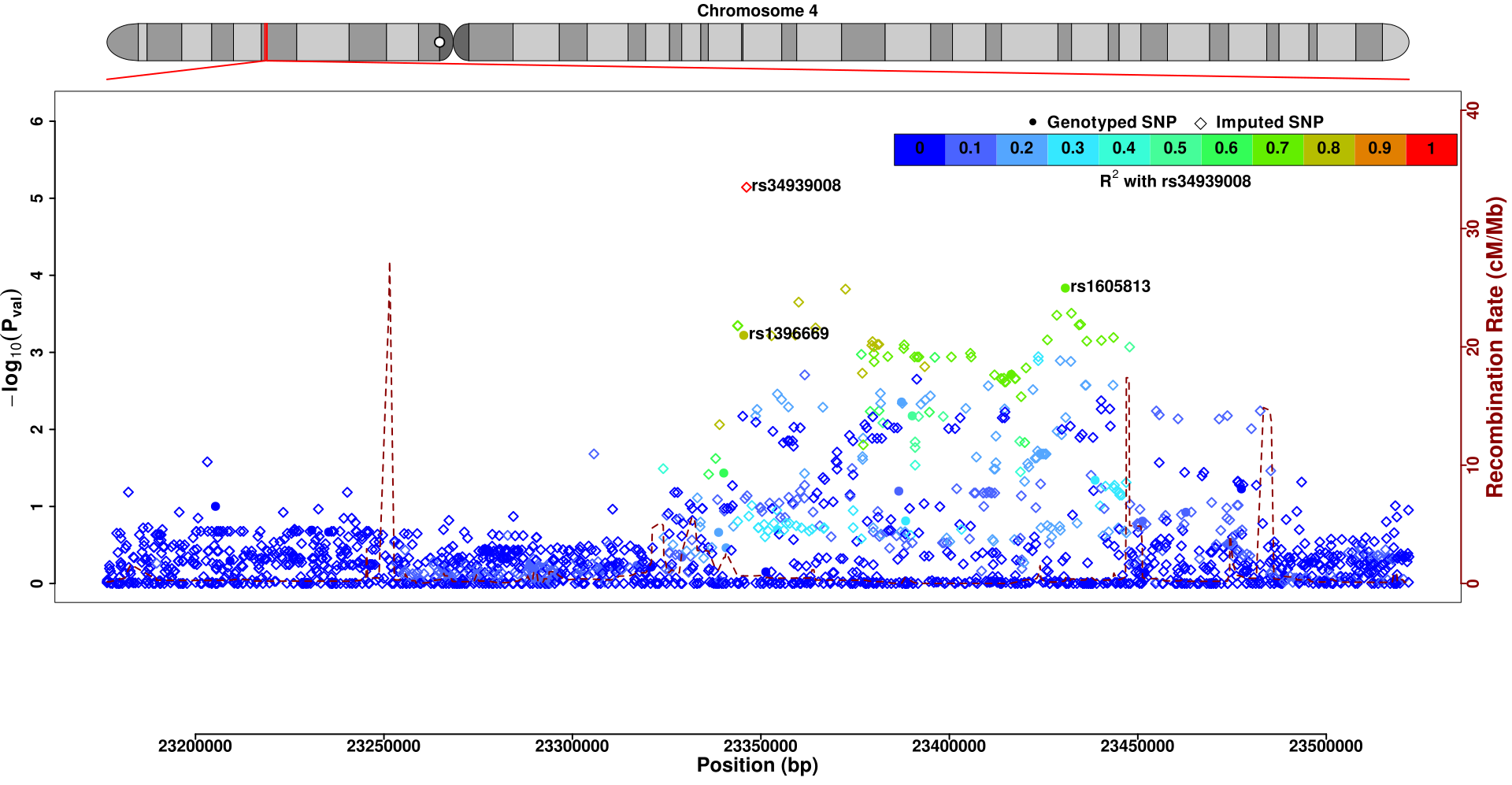

Supplement: S10 Fig — (TIF) [file pgen.1008038.s014.tif]

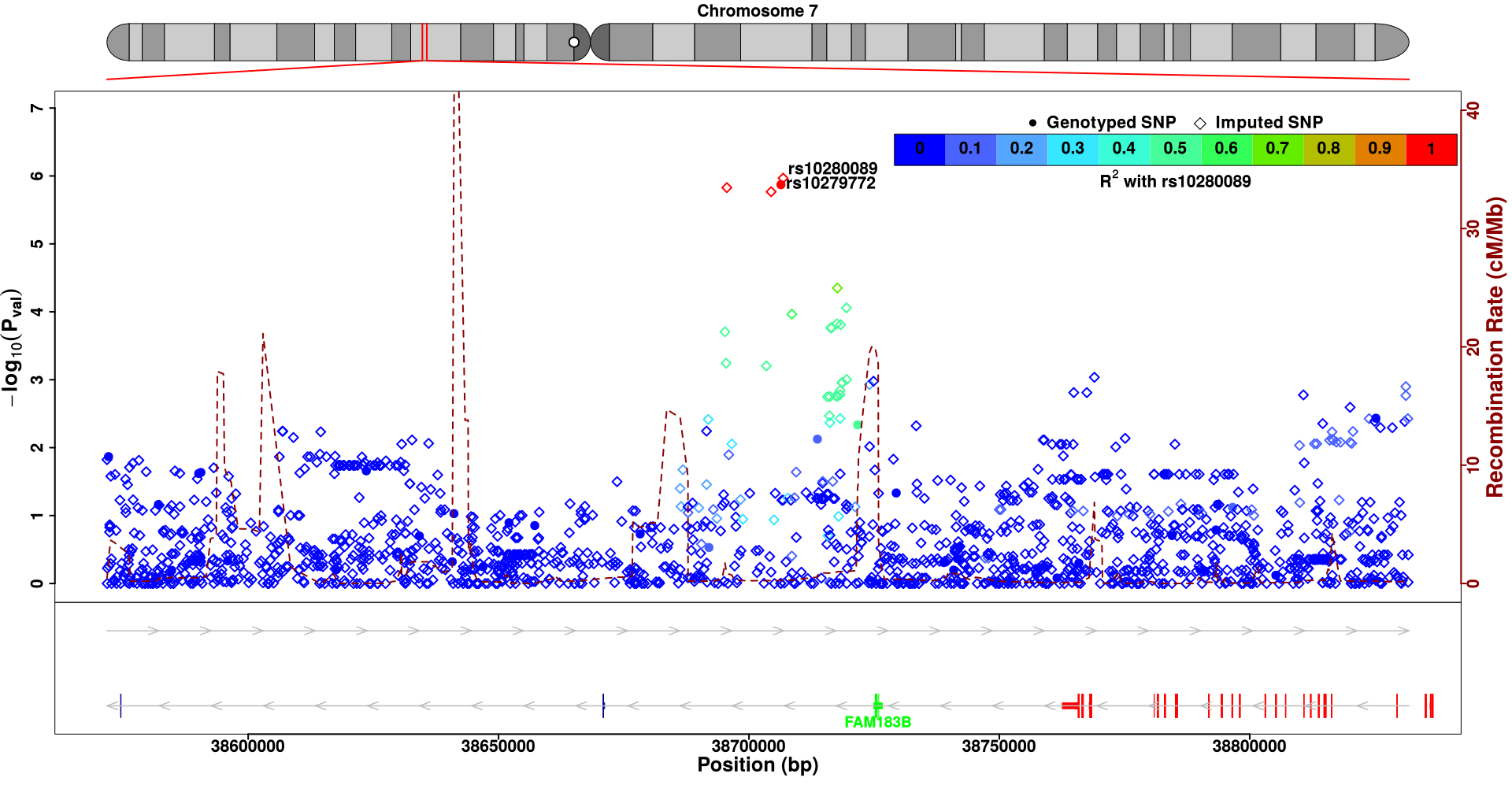

Supplement: S11 Fig — (TIF) [file pgen.1008038.s015.tif]

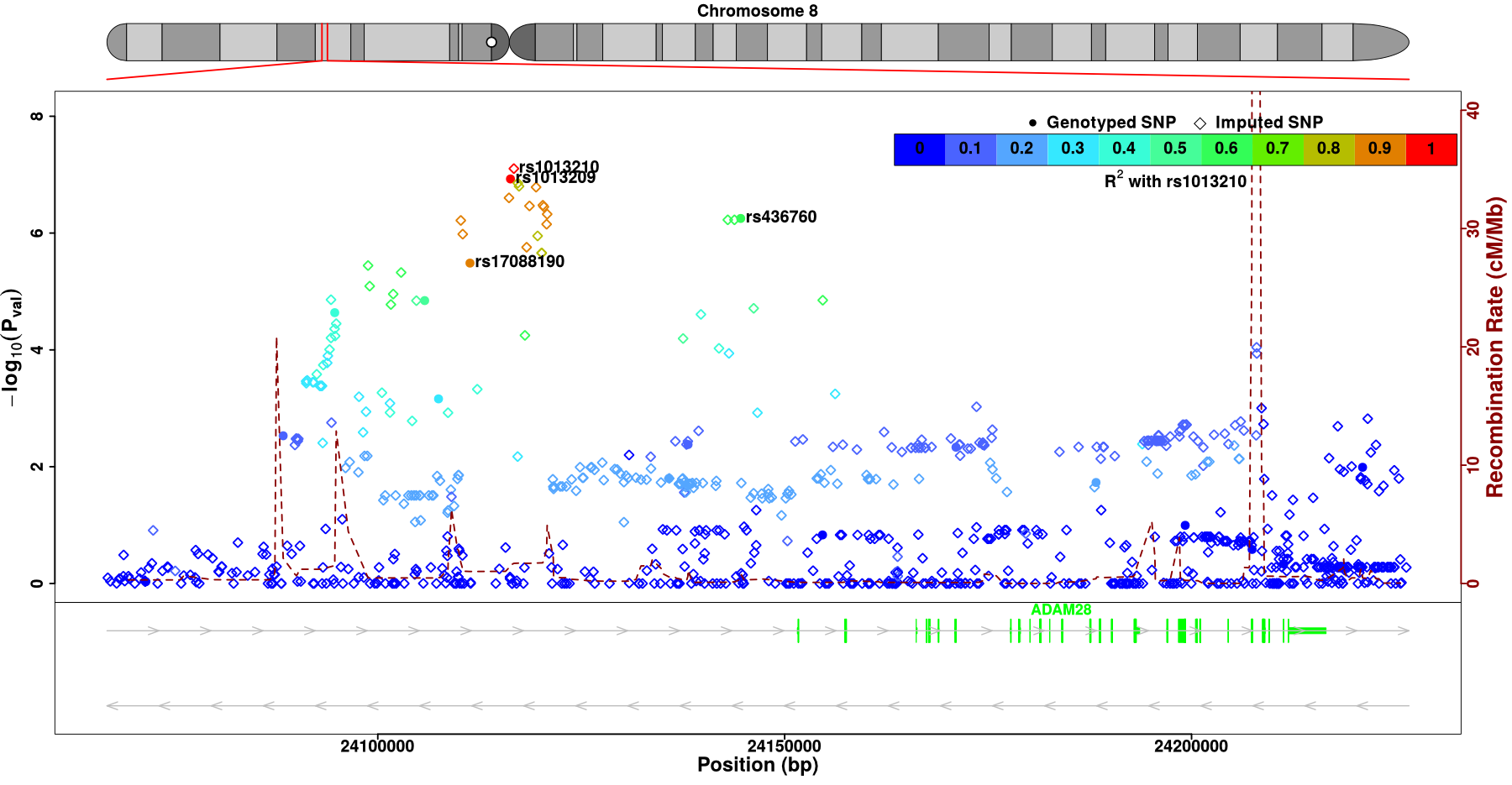

Supplement: S12 Fig — (TIF) [file pgen.1008038.s016.tif]

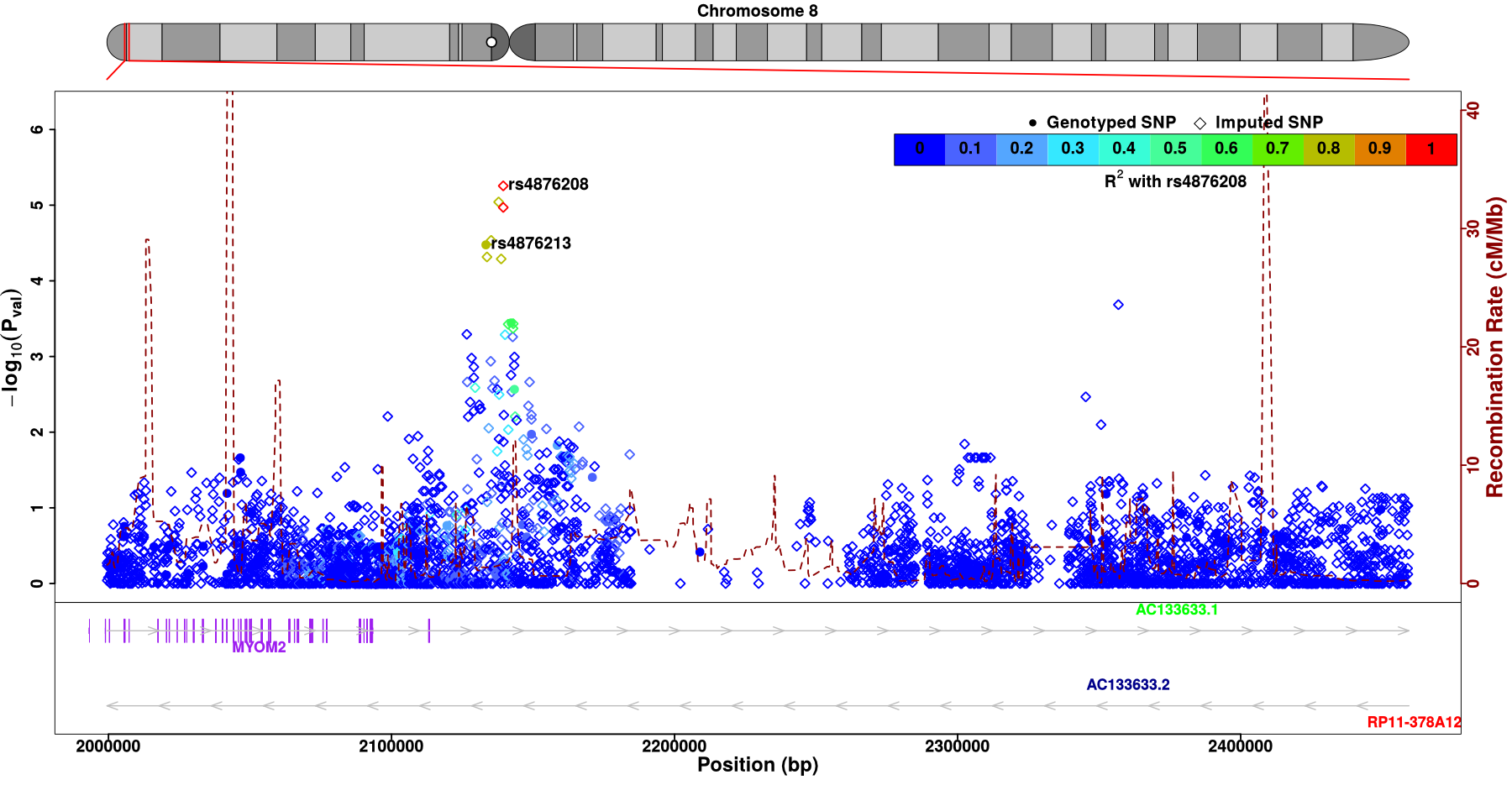

Supplement: S13 Fig — (TIF) [file pgen.1008038.s017.tif]

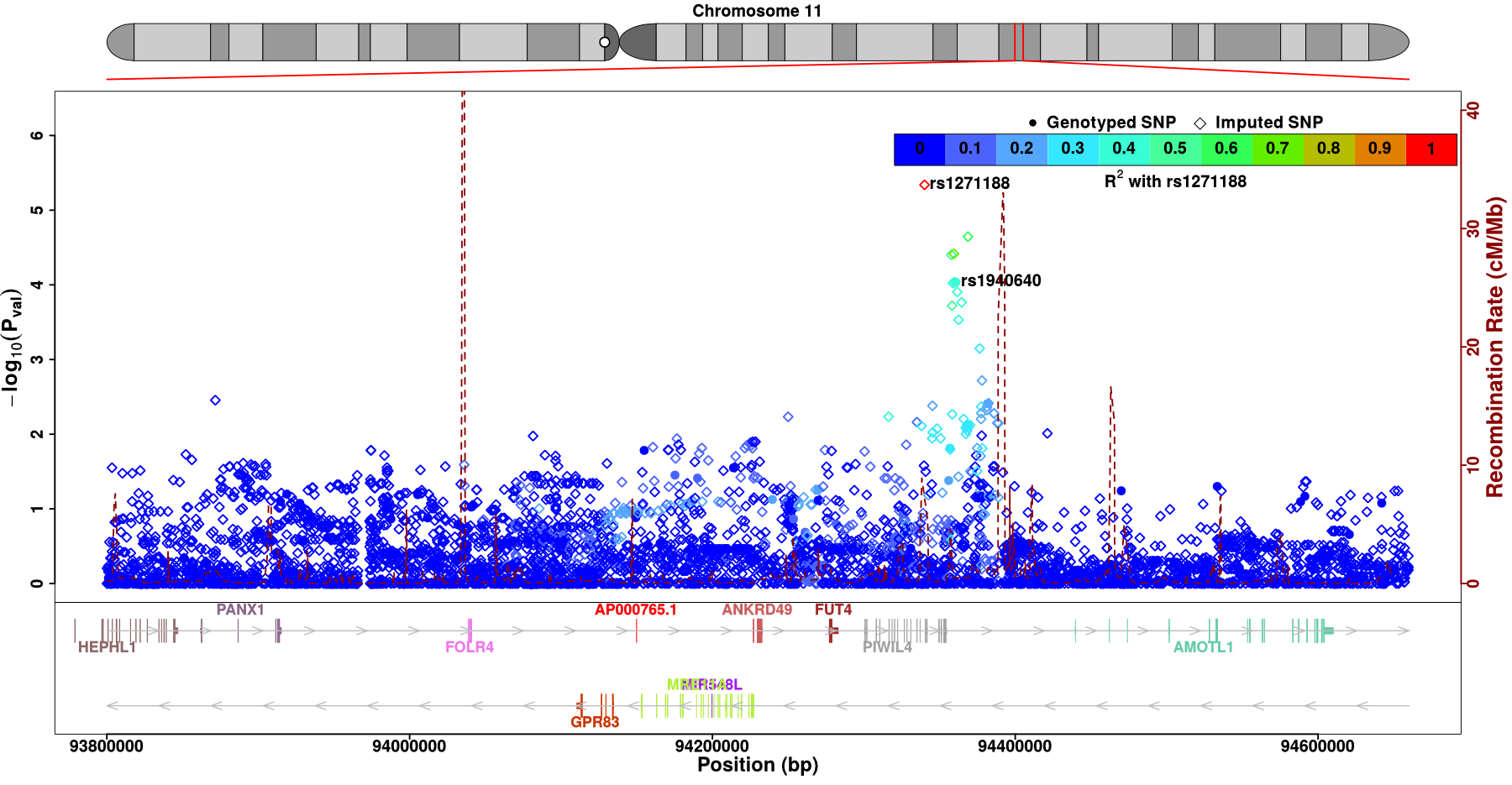

Supplement: S14 Fig — (TIF) [file pgen.1008038.s018.tif]

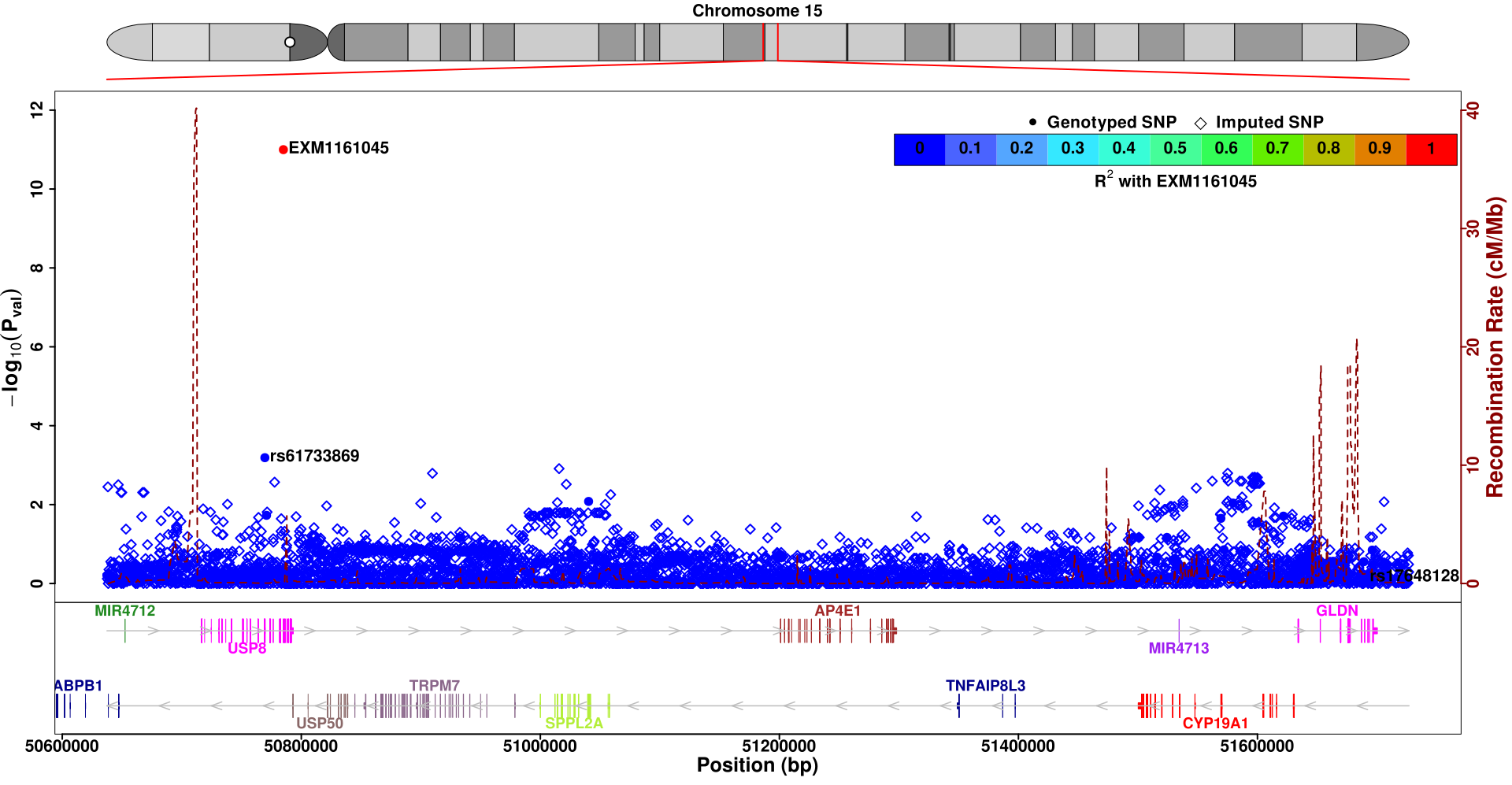

Supplement: S15 Fig — (TIF) [file pgen.1008038.s019.tif]

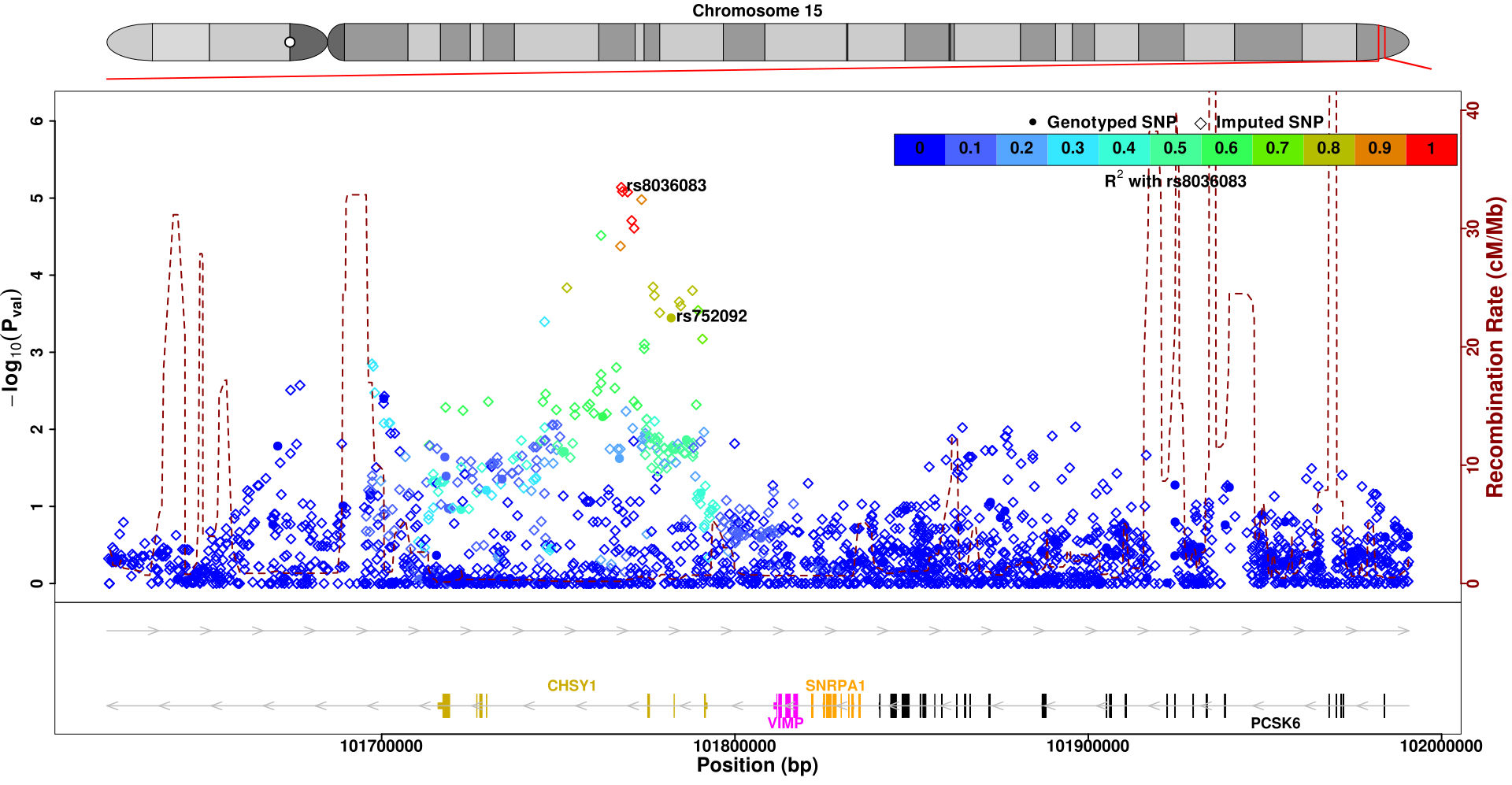

Supplement: S16 Fig — (TIF) [file pgen.1008038.s020.tif]

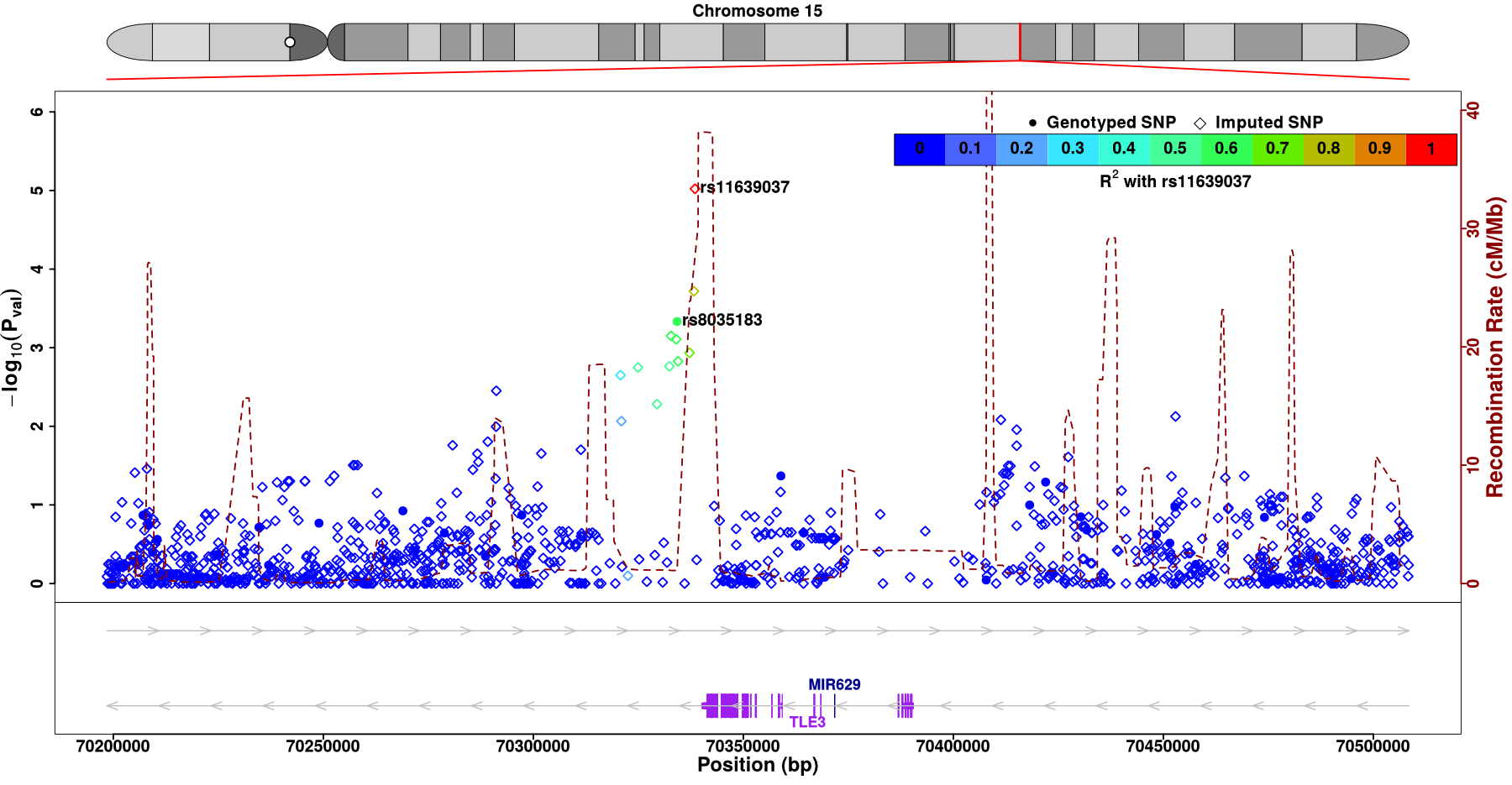

Supplement: S17 Fig — (TIF) [file pgen.1008038.s021.tif]

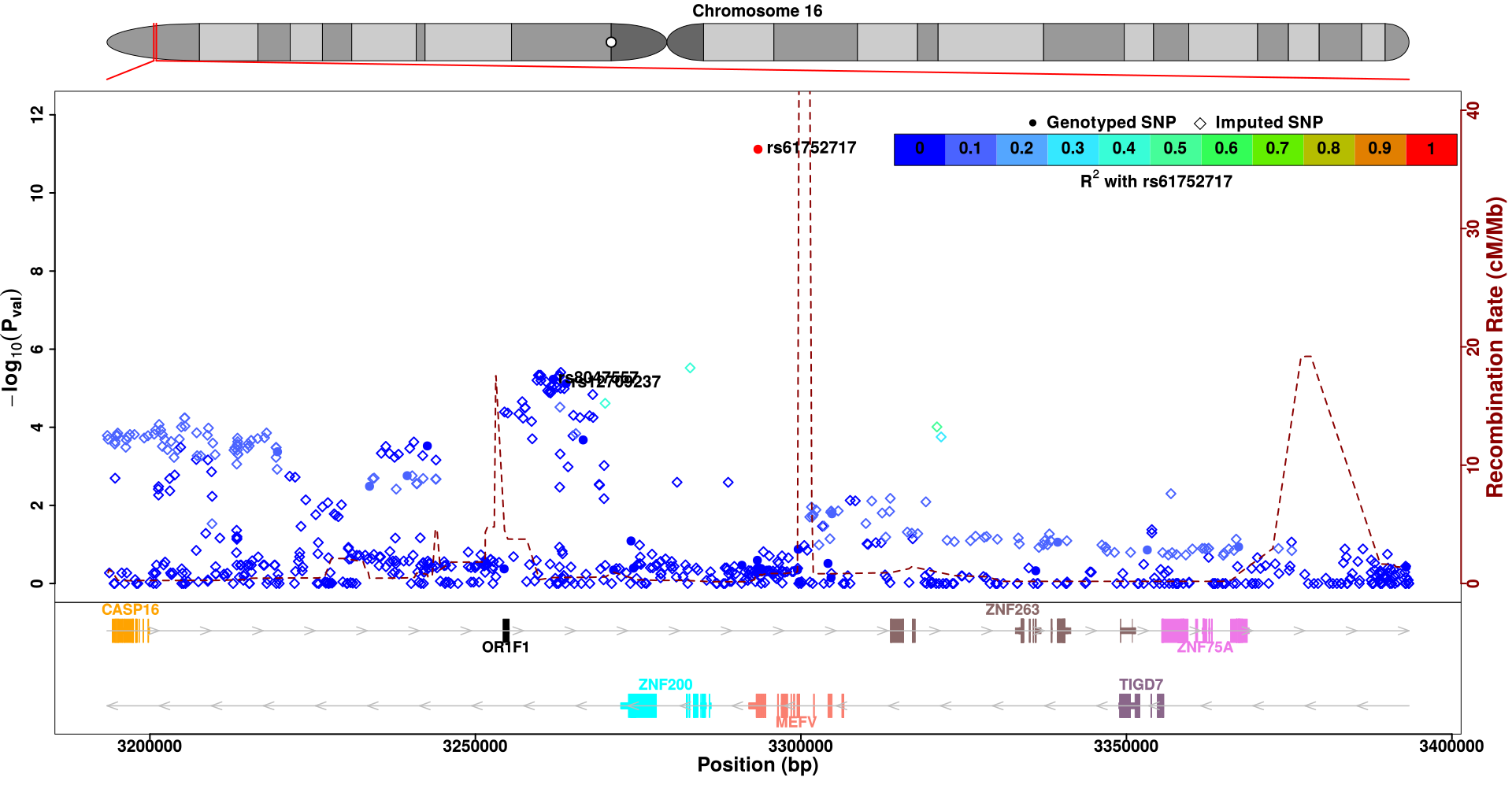

Supplement: S18 Fig — (TIF) [file pgen.1008038.s022.tif]

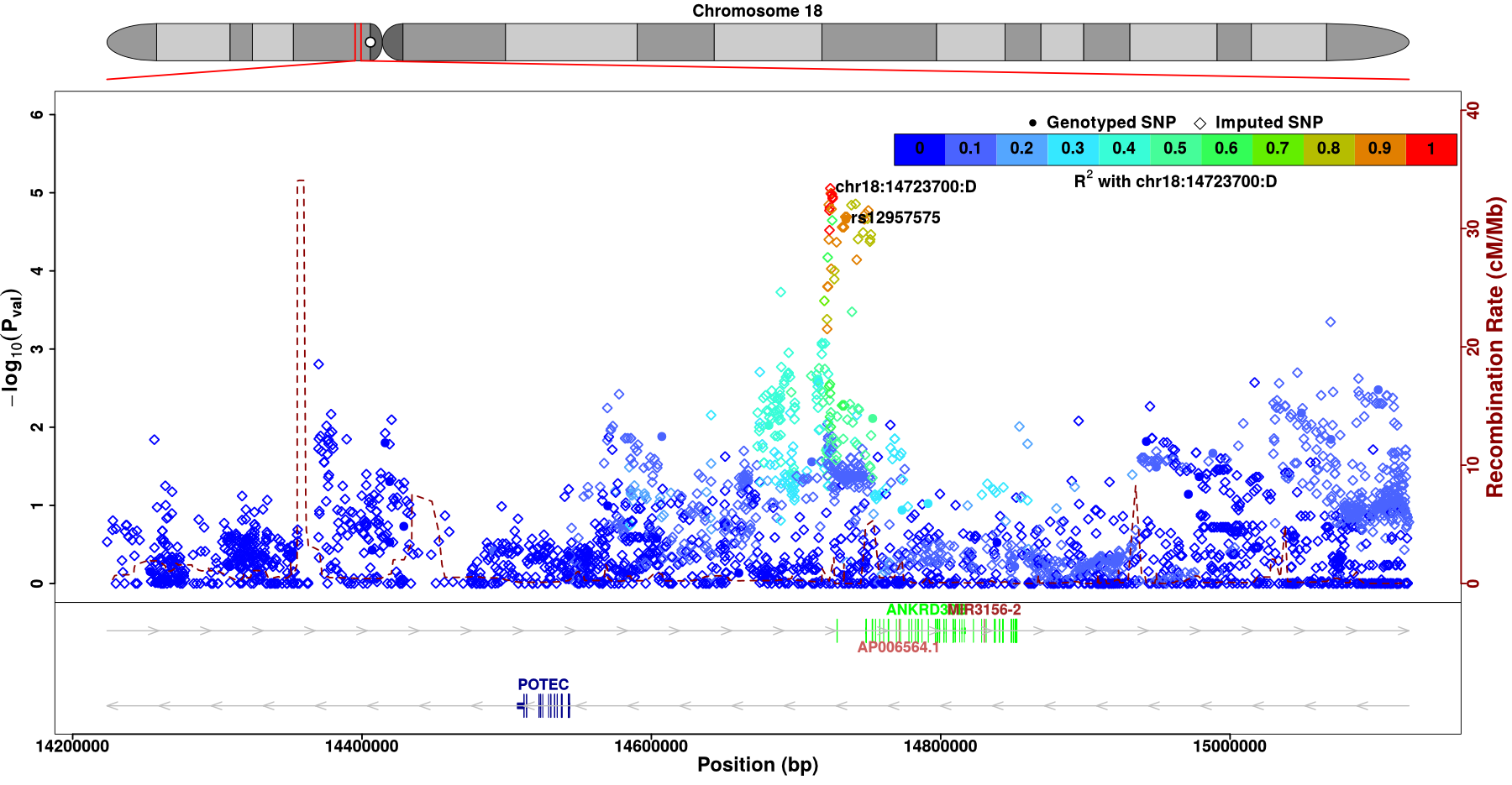

Supplement: S19 Fig — (TIF) [file pgen.1008038.s023.tif]

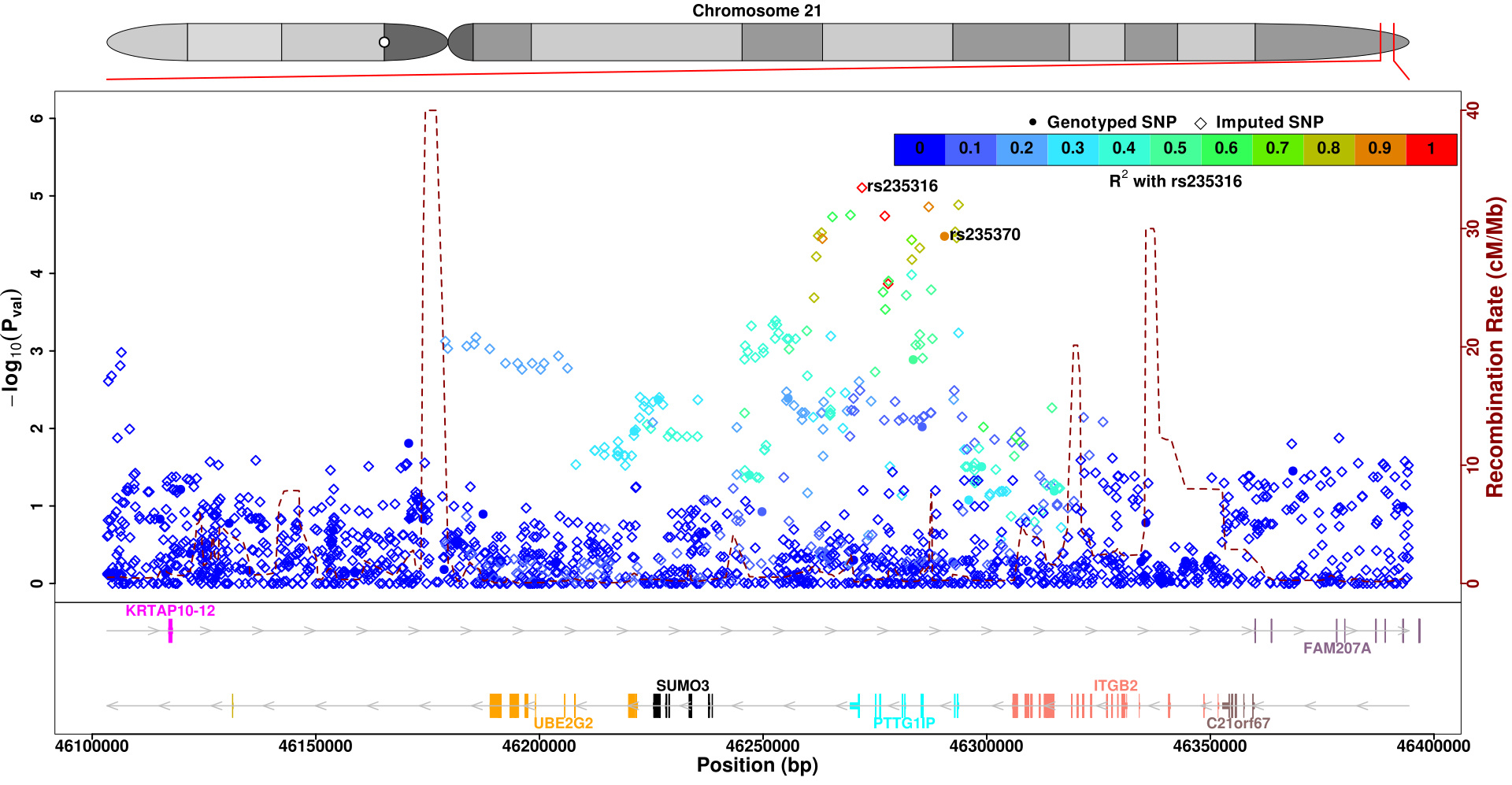

Supplement: S20 Fig — (TIF) [file pgen.1008038.s024.tif]

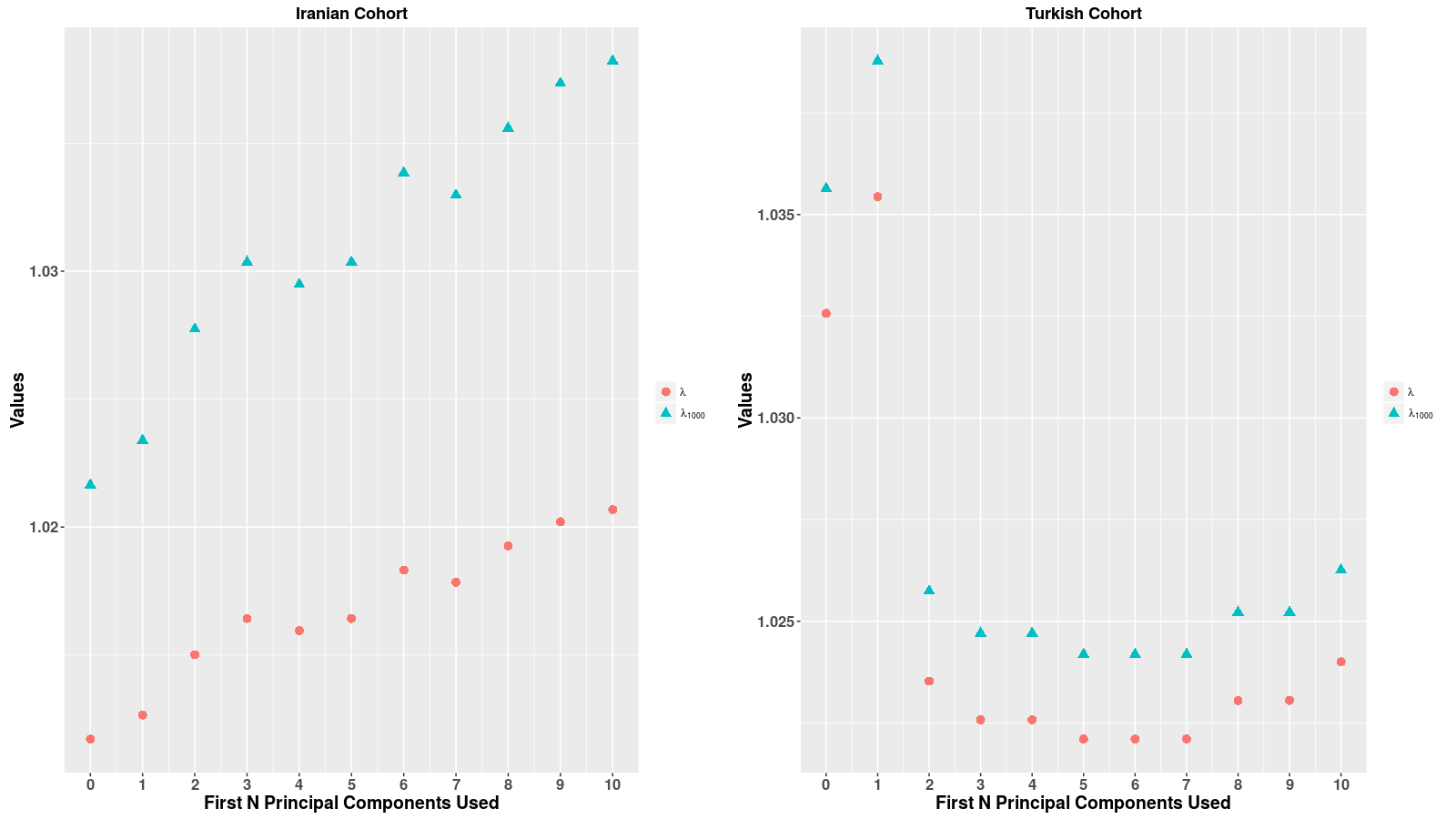

Supplement: S21 Fig — λ and λ1000 are coloured by red and blue, respectively. The x-axis and y-axis are the 1st and corresponding λ and λ1000 values. (TIF) [file pgen.1008038.s025.tif]

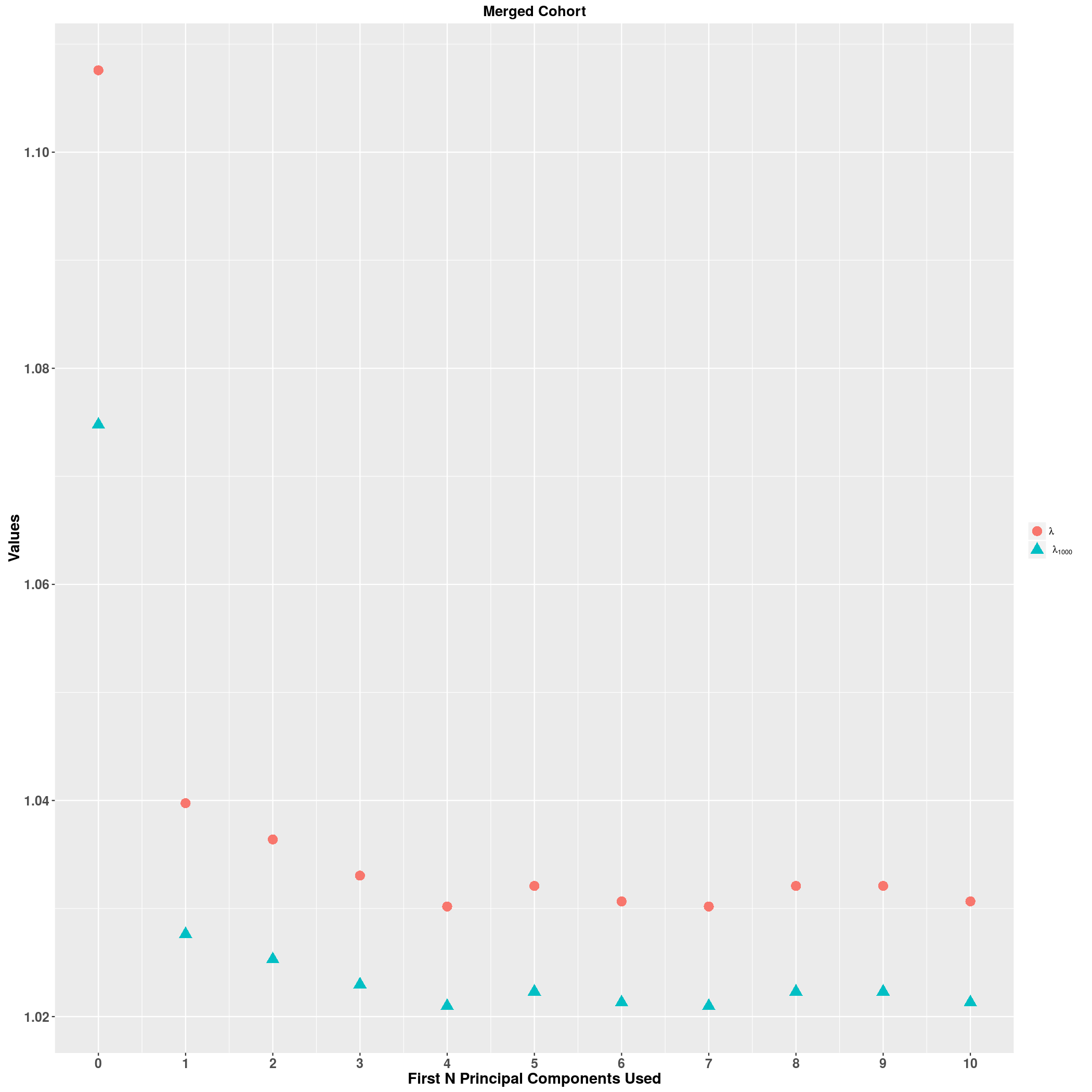

Supplement: S22 Fig — λ and λ1000 are coloured by red and blue, respectively. The x-axis and y-axis are the 1st and corresponding λ and λ1000 values. (TIF) [file pgen.1008038.s026.tif]

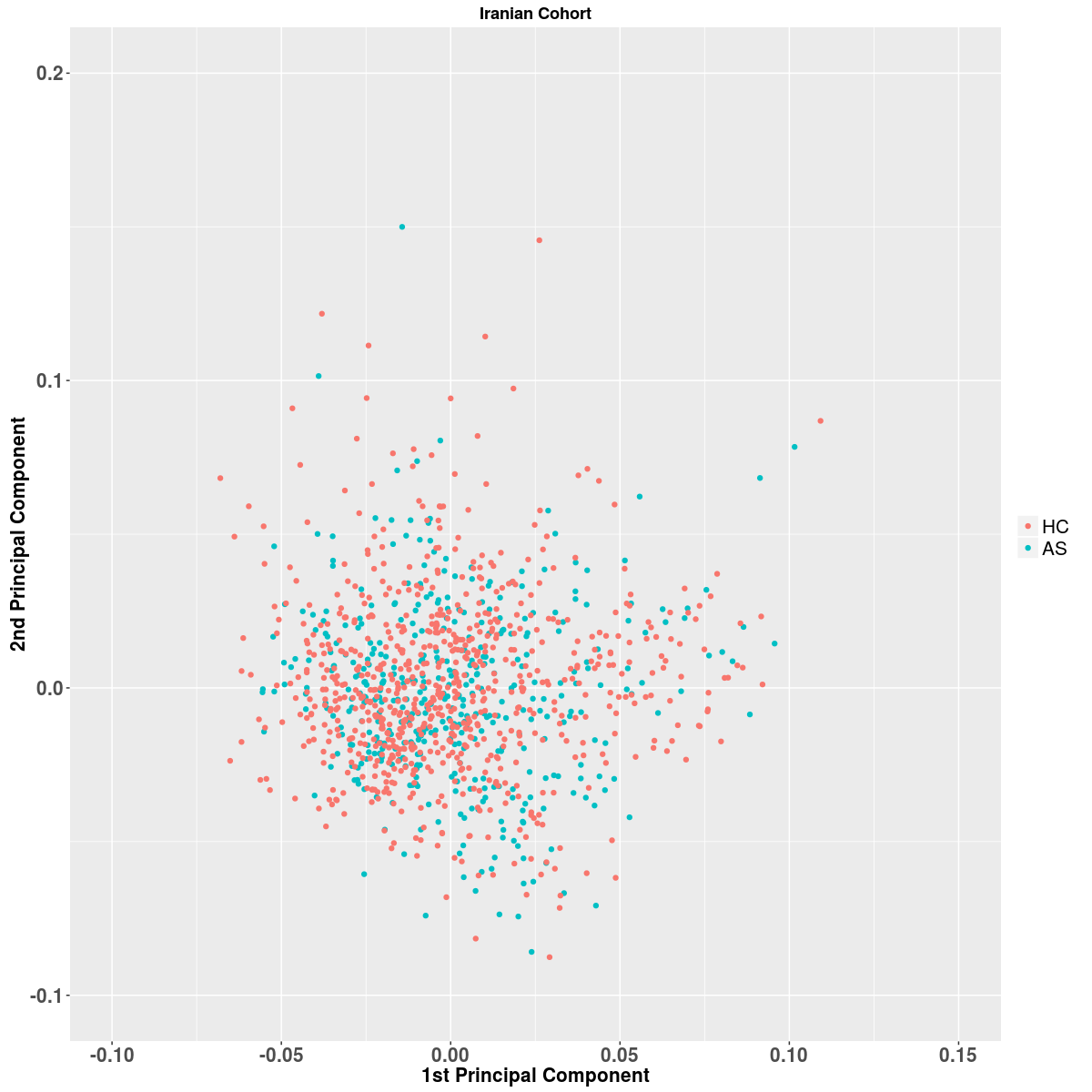

Supplement: S23 Fig — Healthy controls (HC) are in red while ankylosing spondylitis patients (AS) are in blue. The x-axis and y-axis are the 1st and 2nd principal component from the final PCA. (TIF) [file pgen.1008038.s027.tif]

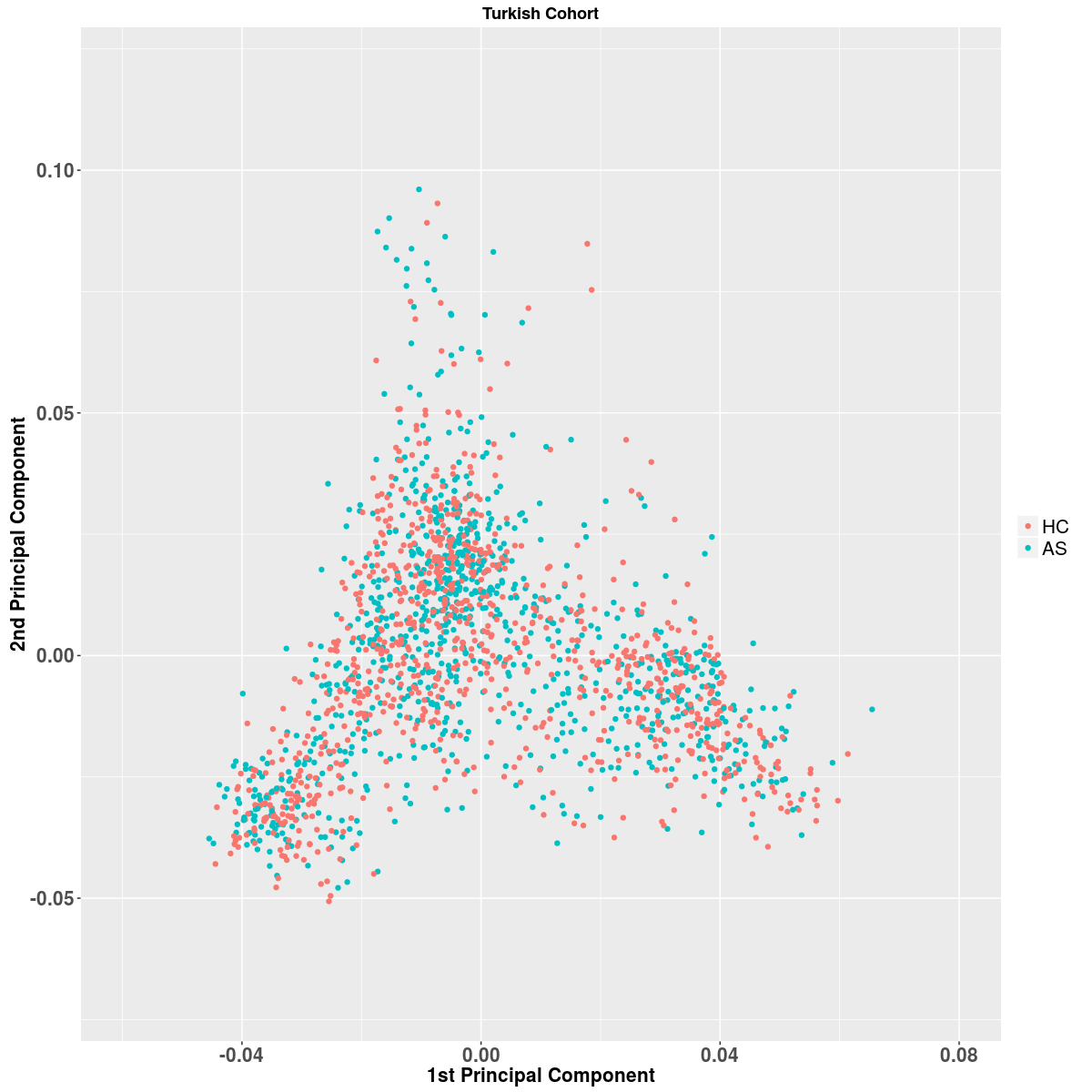

Supplement: S24 Fig — Healthy controls (HC) are in red while ankylosing spondylitis patients (AS) are in blue. The x-axis and y-axis are the 1st and 2nd principal component from the final PCA. (TIF) [file pgen.1008038.s028.tif]

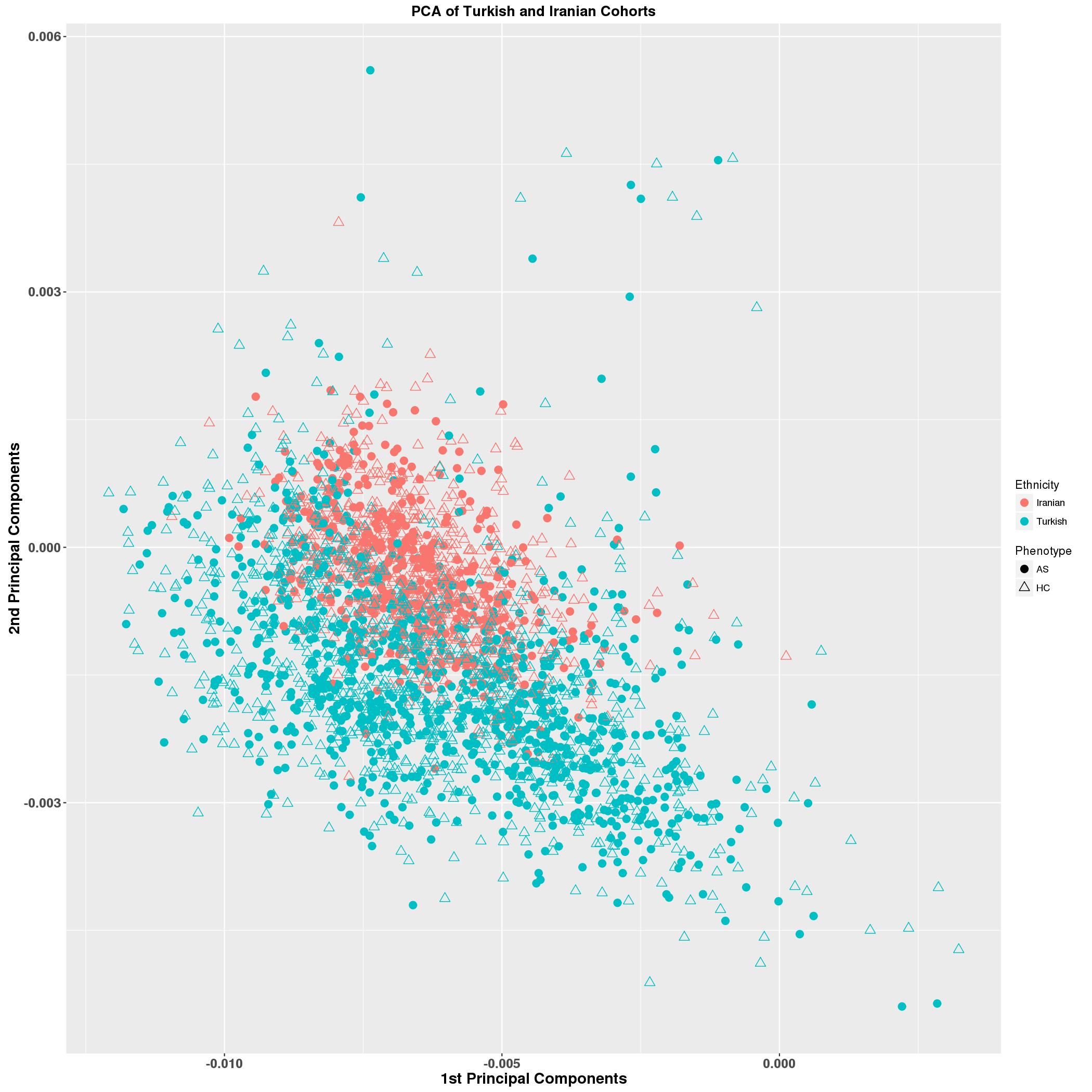

Supplement: S25 Fig — Iranian samples are in red while Turkish samples are in blue. The ankylosing spondylitis (AS) and healthy control (HC) are indicated by solid dots and triangles, respectively. The x-axis and y-axis are the 1st and 2nd principal component from the final PCA. (TIF) [file pgen.1008038.s029.tif]

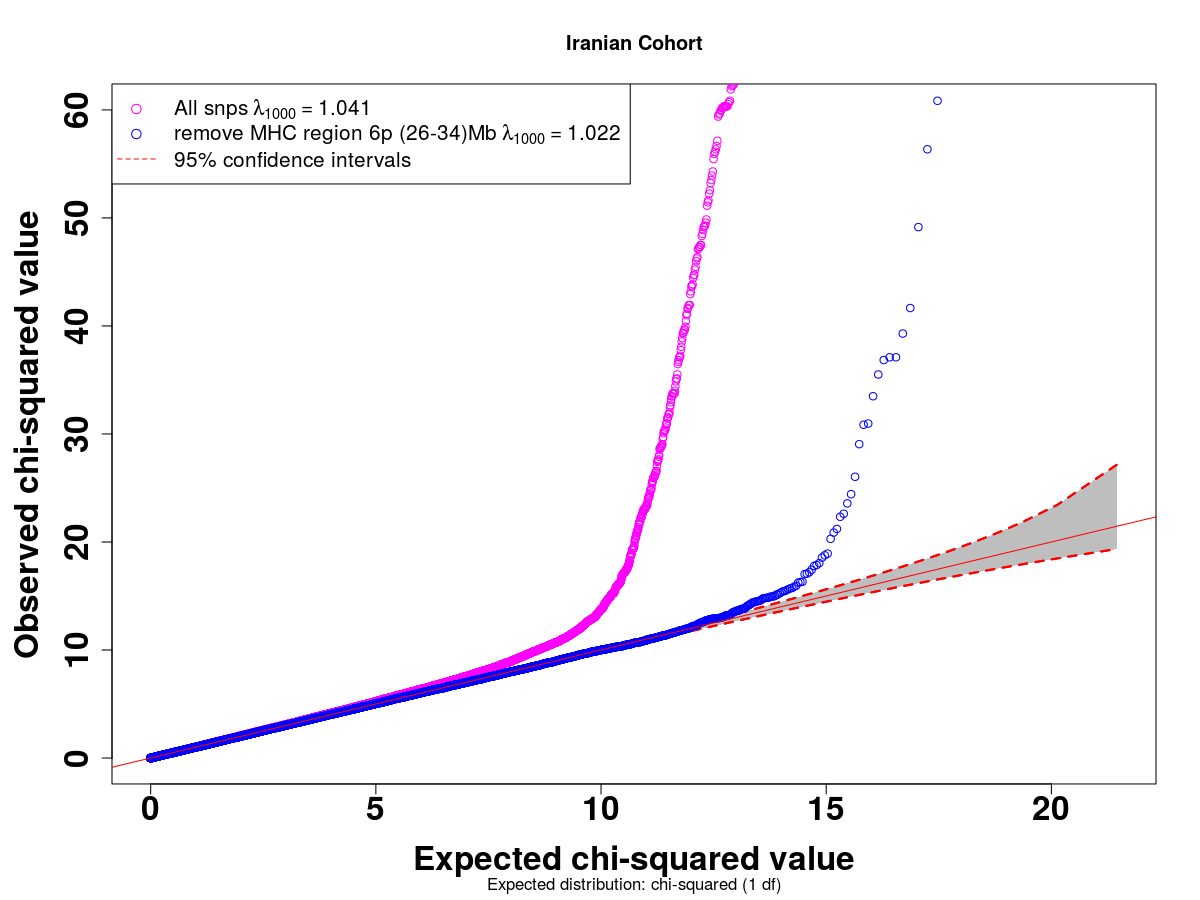

Supplement: S26 Fig — Red dashed lines indicate 95% CI of expected χ2 values, while magenta and blue points are the observed χ2 values of the entire dataset and the dataset excluding the MHC region, respectively. (TIF) [file pgen.1008038.s030.tif]

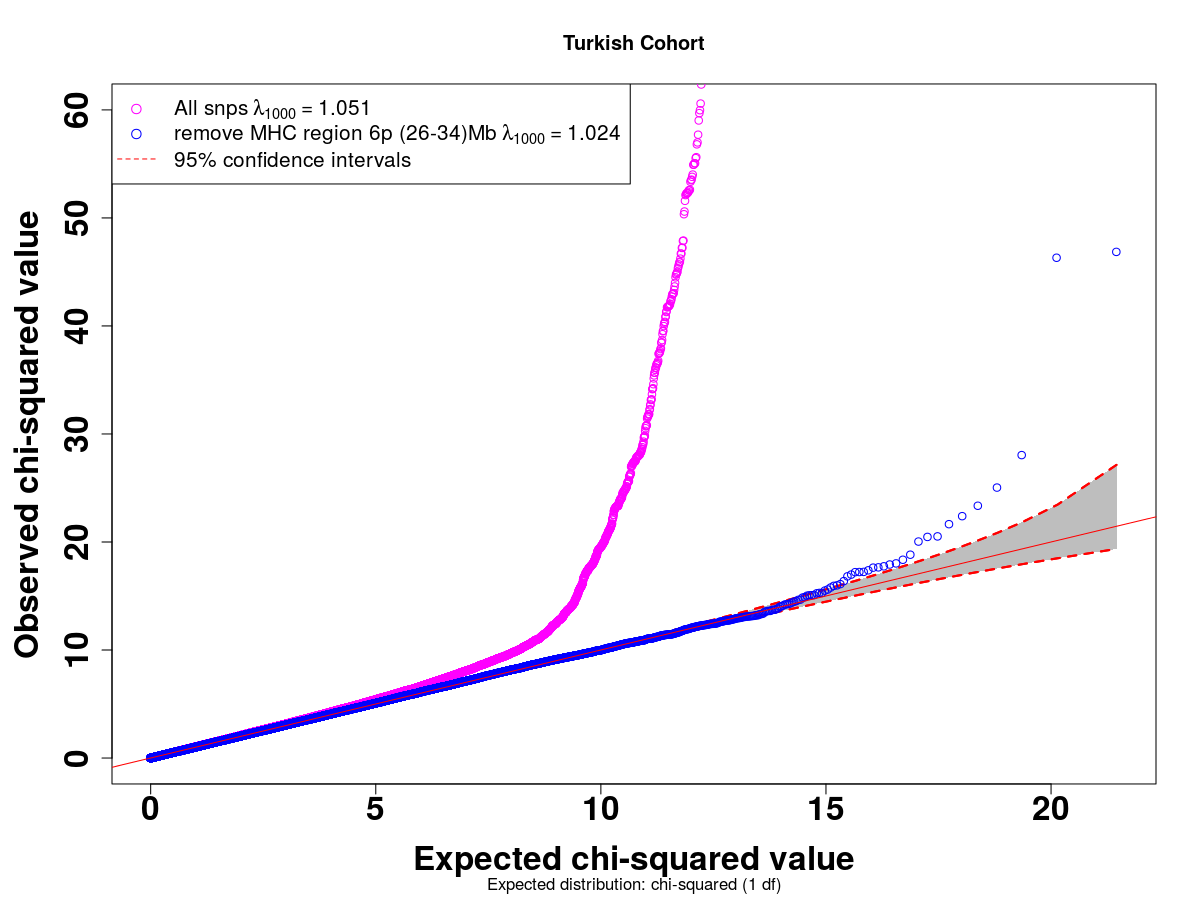

Supplement: S27 Fig — The λ1000 was 1.051 for overall dataset and 1.024 excluding MHC region. Red dashed lines indicate 95% CI of expected χ2 values, while magenta and blue points are the overserved χ2 values of the entire dataset and dataset excluding the MHC region, respectively. (TIF) [file pgen.1008038.s031.tif]

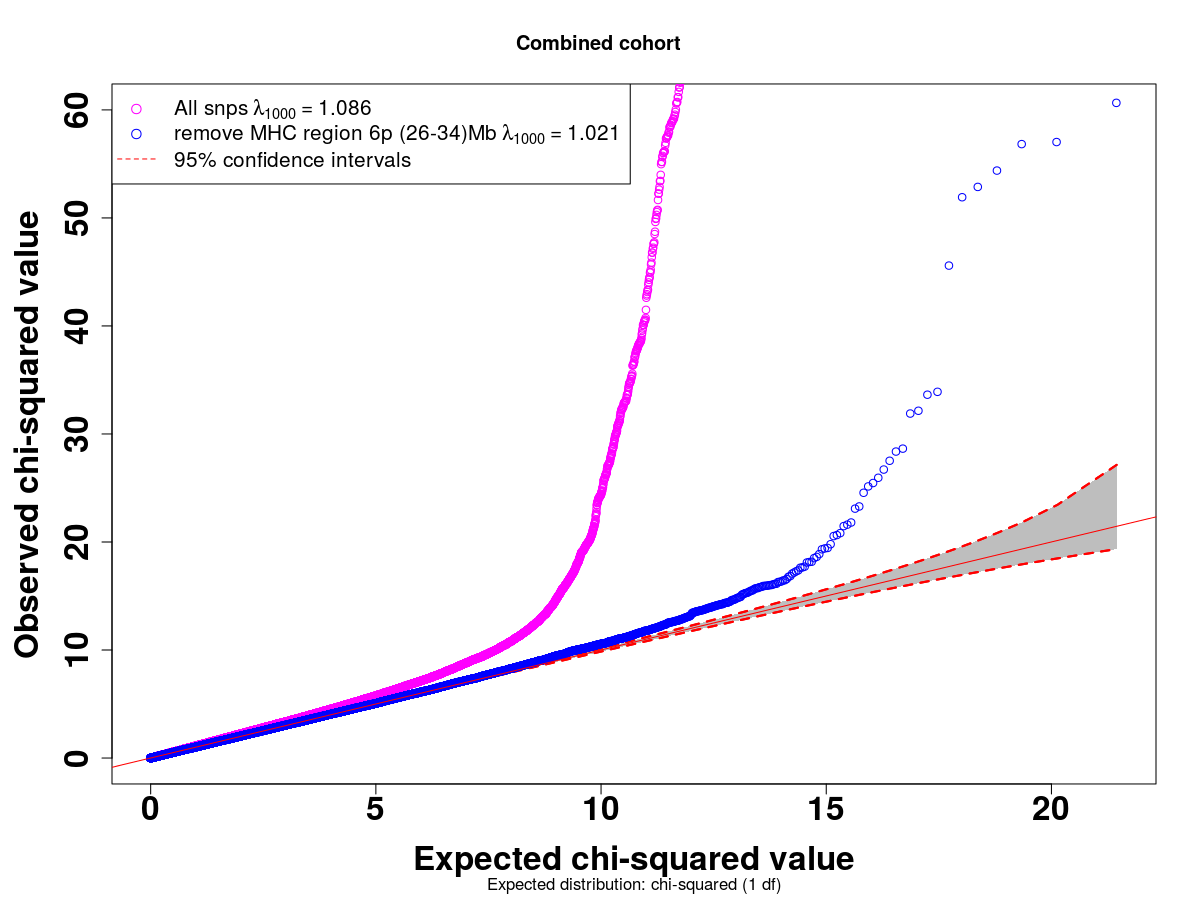

Supplement: S28 Fig — The λ1000 was 1.086 for the entire dataset and 1.021 excluding the MHC region. Red dashed lines indicate 95% CI of expected χ2 values, while magenta and black points are the overserved χ2 values of the entire dataset and dataset excluding the MHC region, respectively. (TIF) [file pgen.1008038.s032.tif]
